# Integrating complementary methods to improve diet analysis in fishery‐targeted species

**DOI:** 10.1002/ece3.4456

**Published:** 2018-08-29

**Authors:** Jordan K. Matley, Gregory E. Maes, Floriaan Devloo‐Delva, Roger Huerlimann, Gladys Chua, Andrew J. Tobin, Aaron T. Fisk, Colin A. Simpfendorfer, Michelle R. Heupel

**Affiliations:** ^1^ Center for Marine and Environmental Studies University of the Virgin Islands St. Thomas Virgin Islands; ^2^ Great Lakes Institute for Environmental Research University of Windsor Windsor Ontario Canada; ^3^ Laboratory of Biodiversity and Evolutionary Genomics KU Leuven Leuven Belgium; ^4^ Center for Human Genetics UZ Leuven‐ Genomics Core KU Leuven Leuven Belgium; ^5^ Centre for Sustainable Tropical Fisheries and Aquaculture College of Science and Engineering James Cook University Townsville Queensland Australia; ^6^ Comparative Genomics Centre College of Science and Engineering James Cook University Townsville Queensland Australia; ^7^ Oceans and Atmosphere CSIRO Hobart Tasmania Australia; ^8^ Australian Institute of Marine Science Townsville Queensland Australia

**Keywords:** coral reef, coral trout, diet, fisheries, metabarcoding, next‐generation sequencing, *Plectropomus*, stable isotopes, stomach contents

## Abstract

Developing efficient, reliable, cost‐effective ways to identify diet is required to understand trophic ecology in complex ecosystems and improve food web models. A combination of techniques, each varying in their ability to provide robust, spatially and temporally explicit information can be applied to clarify diet data for ecological research. This study applied an integrative analysis of a fishery‐targeted species group—*Plectropomus* spp. in the central Great Barrier Reef, Australia, by comparing three diet‐identification approaches. Visual stomach content analysis provided poor identification with ~14% of stomachs sampled resulting in identification to family or lower. A molecular approach was successful with prey from ~80% of stomachs identified to genus or species, often with several unique prey in a stomach. Stable isotope mixing models utilizing experimentally derived assimilation data, identified similar prey as the molecular technique but at broader temporal scales, particularly when prior diet information was incorporated. Overall, Caesionidae and Pomacentridae were the most abundant prey families (>50% prey contribution) for all *Plectropomus* spp., highlighting the importance of planktivorous prey. Less abundant prey categories differed among species/color phases indicating possible niche segregation. This study is one of the first to demonstrate the extent of taxonomic resolution provided by molecular techniques, and, like other studies, illustrates that temporal investigations of dietary patterns are more accessible in combination with stable isotopes. The consumption of mainly planktivorous prey within this species group has important implications within coral reef food webs and provides cautionary information regarding the effects that changing resources could have in reef ecosystems.

## 1 INTRODUCTION

Prey acquisition is a fundamental biological process that drives development and behavior (e.g., growth, reproduction, foraging) of individuals, and contributes to population‐level characteristics (e.g., migration, trophic position, habitat selection). Prey selection and availability can also have ongoing and multiplicative ecological effects within an ecosystem (e.g., trophic cascades; Estes et al., 2011) because consumers are often resource‐limited or have overlapping dietary preferences (Ross, 1986; Sale, 1977). Empirical diet data help quantify the relative importance of prey items and characterize ecological interactions (e.g., resource partitioning, trophodynamics, competition) that occur within and among species (Connell, 1980; Schoener, 1974).

For fishes, there are several ways to identify or quantify diet. There are also several considerations in selecting methods to characterize diet. These vary on a case‐by‐case basis and the goals of the research, but are constrained by the cost of approach, lethal vs nonlethal sampling, number of samples/individuals required, necessity of repeat sampling, and/or resolution provided by approach (e.g., temporal or identification resolution). One of the most direct methods is a visual examination of identifiable prey from stomach contents, and while this provides a snapshot of feeding (e.g., hours‐days), digestion limits identification, stomachs are often empty, and large sample sizes and lethal sampling are generally required (St John, 1999; Vinson & Budy, 2011). However, advances in molecular approaches provide a potential alternative to visual stomach content analysis (Carreon‐Martinez, Johnson, Ludsin, & Heath, 2011; Leray, Meyer, & Mills, 2015). The ability to sequence prey items from degraded stomach contents enhances diet data and has the capacity to reduce inefficiencies caused by unidentifiable samples. Nevertheless, this metabarcoding approach is still limited by the completeness of reference sequence databases and the choice of genetic markers (Devloo‐Delva et al., 2018); consequently, prior validation is needed for newly studied species/systems. Another method to characterize diet is stable isotope analysis (e.g., δ^15^N and δ^13^C), a biogeochemical indicator of prey assimilation in the tissues of consumers (see Newsome, Clementz, & Koch, 2010 for review). Due to different metabolic processing within tissues, the timeline (or turnover) representing prey assimilation varies depending on the tissue sampled. For example, Matley, Fisk, Tobin, Heupel, and Simpfendorfer (2016) found that 50% incorporation times (or 50% turnover) of δ^15^N in plasma, red blood cells (RBC), and muscle tissues of the predatory coral reef fish *Plectropomus leopardus*, were 66, 88, and 126 days, respectively. As δ^15^N and δ^13^C values change from prey to consumer by conserved amounts, the identity (e.g., species, family, habitat) and relative importance of different prey sources can be estimated (e.g., mixing models; Chiaradia, Forero, McInnes, & Ramírez, 2014). This approach requires methodical sampling of potential prey items, and standardization of assimilation parameters (e.g., diet‐tissue discrimination factors) that may not exist for that species; thus, it often requires additional sampling/testing over other methods. Stable isotopes also reflect assimilation patterns of often confounding dietary sources over relatively long periods of time and therefore is a representation of broad‐scale patterns (i.e., does not necessarily identify exact prey) over the temporal scale pertinent to the tissue sampled. Each method for analyzing diet includes limitations; a combination of approaches has the potential to provide greater resolution and clarity at multiple spatial and temporal scales.

The first objective of this study was to compare three dietary sampling approaches (i.e., visual, genetic, stable isotope analysis) to identify the advantages and weaknesses of each technique in isolation and combined. A congeneric group of coral trout (*Plectropomus* spp.), were selected because they are widespread mesopredators found throughout the Indo‐Pacific with significant fishery value (Sadovy de Mitcheson et al., 2013). Multiple past studies using visual stomach content analysis have shown that the diet of adult *P. leopardus*, the most abundant *Plectropomus* species in the Great Barrier Reef Marine Park (GBRMP) in Australia, consists of >25 prey families, but is mainly comprised of Clupeidae, Pomacentridae, and Labridae (Kingsford, 1992; St John, 1999). Dietary comparisons between sympatric *Plectropomus* are of interest because they can reflect competitive interactions or niche partitioning, which can help elucidate small‐scale distributional patterns and capacity for hybridization (e.g., Harrison et al., 2017). However, dietary comparisons between sympatric *Plectropomus* are scarce; isotopic (δ^15^N and δ^13^C) niche differed between *P. laevis* and *P. leopardus* (Matley, Tobin, Simpfendorfer, Fisk, & Heupel, 2017), and *P. maculatus* and *P. leopardus* (Frisch, Ireland, & Baker, 2014) at reefs off Townsville and Northwest Island, respectively. However, isotopic niche between *P. maculatus* and *P. leopardus* was similar at Orpheus Island Reef (Matley, Heupel, Fisk, Simpfendorfer, & Tobin, 2016). Examination of stomach content has yet to be completed for *Plectropomus* species in sympatry. Therefore, the second objective of this study was to identify and quantify the composition of prey consumed by *Plectropomus* spp. to explore niche segregation and further inform on prey consumption patterns of an iconic species group.

## 2 MATERIALS AND METHODS

### 2.1 Sample collection

**Figure 1:**
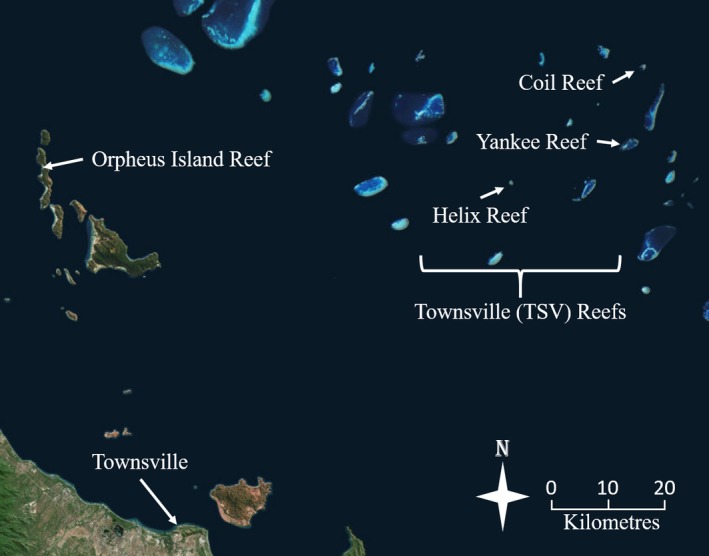
Locations of coral trout collection for stable isotopes and DNA gut contents. *Plectropomus leopardus* was sampled at all locations, *P. maculatus* was sampled at Orpheus Island (OI) Reef, and *P. laevis* was sampled at Coil Reef, Yankee Reef, and Helix Reef within the Townsville (TSV) sector of the Great Barrier Reef Marine Park. The city of Townsville is identified for reference

Three species of *Plectropomus* were collected within the GBRMP between August 2013 and May 2014 for visual, molecular, and stable isotope diet analysis (Table 1). *Plectropomus leopardus* (*n* = 90; mean ± *SE*: 455 ± 6 mm; range: 276–577 mm) and *P. laevis* (*n* = 36; mean ± *SE*: 522 ± 24 mm; range: 299–910 mm) were collected at midshelf reefs off Townsville, Australia (TSV: Helix Reef, Yankee Reef, Coil Reef; Figure 1), and *P. leopardus* (*n* = 9; mean ± *SE*: 475 ± 16 mm; range: 377–610 mm) and *P. maculatus* (*n* = 10; mean ± *SE*: 358 ± 20 mm; range: 280–515 mm) were sampled at Orpheus Island (OI) Reef—an inshore reef on west side of Orpheus Island (Figure 1). Individuals were taken by speargun while diving with SCUBA (<20 m deep).

**Table 1:** Summary of *Plectropomus* spp. sample collection for visual and DNA stomach content analysis, and stable isotope analysis (SIA)

Species	Reef	Date	Visual stomach contents (*n*)	DNA stomach contents (*n*)	SIA plasma (*n*)	SIA RBC (*n*)	SIA muscle (*n*)
*P. leopardus*	Helix	August 2013	11 (2)	10 (8)	0	0	11
*P. laevis* (footballer)	Coil, Helix, Yankee	November 2013	8 (1)	10 (10)	6	7	8
*P. laevis* (bluespot)	Coil, Helix, Yankee	November 2013	28 (4)	23 (22)	28	27	28
*P. leopardus*	Coil, Helix, Yankee	November 2013	58 (8)	35 (21)	39	49	58
*P. leopardus*	Helix	February 2014	21 (4)	3 (2)	19	20	21
*P. leopardus*	Orpheus	May 2014	10 (3)	10 (9)	0	0	9
*P. maculatus*	Orpheus	May 2014	10 (4)	10 (9)	0	0	10

*Note*

Brackets represent the number of samples where prey were identified

### 2.2 Visual stomach content identification

Stomachs were removed upon collection and frozen (−20°C). Stomachs were thawed, dissected, and prey items classified based on the digestion level (1–4 = low–high digestion: 1—little or no digestion except superficially, for example, skin and fins; 2—moderate digestion with head and tail mostly digested and possibility of parts broken off and oval fleshy remains; 3—major digestion with small fleshy remains and abundance of broken parts; 4—complete digestion with very small fragments of prey remaining or empty stomach and clean lining). Prey (digestion level 1 and 2) were weighed (0.001 g) and identified to the lowest taxonomical level possible using Randall, Allen, and Steene (1997) and Froese and Pauly (2016). An additional 81 stomachs were collected for visual stomach content identification: Lodestone Reef (16—*P. leopardus* in Nov 2013; and 2—*P. laevis* footballer, 11—*P. leopardus* in Feb 2014), Keeper Reef (1—*P. laevis* footballer, 17—*P. leopardus* in Aug 2013), Centipede Reef (16—*P. leopardus* in Aug 2013), and Wheeler Reef (17—*P. leopardus* in Nov 2013). These samples were not used for metabarcoding and stable isotopes, but are presented here to further evaluate the success of prey identification via this method.

### 2.3 Diet metabarcoding

Stomach contents from each individual were homogenized and DNA extracted following the CTAB protocol from Tamari and Hinkley (2016). Devloo‐Delva et al. (2018) established the method's ability for prey diversity recovery in *Plectropomus* spp., using cytochrome oxidase I primers (mlCOIintF/jgHCO2198; Leray et al., 2013). Amplicon polymerase chain reactions (PCR) were completed with this primer set in a 20 μl reaction volume with 1 μl of template DNA, 1X MyTaq reaction buffer (Bioline, UK), 0.4 μM tailed forward and reverse primer with 10% untailed primers (to initiate amplification), and 0.05 u/μl MyTaq DNA polymerase (Bioline). PCR amplification was performed on a C1000 Thermo Cycler (BIO‐RAD, USA). PCR conditions were set to initial denaturation of 60 s at 95°C, then 40 cycles of 30 s denaturation at 95°C, annealing at 56°C for 30 s, and an extension at 72°C for 30 s. Next, PCR products were indexed using the Nextera Index Kit A (Illumina, USA). In a final volume of 50 μl, we used 5 μl of amplicon PCR product, 1X MyTaq reaction buffer (Bioline), 5 μl of each indexing primer and 0.05 u/μl MyTaq DNA polymerase (Bioline). After each PCR step, products were cleaned using the serapure beads protocol (Faircloth & Glenn, 2014; Rohland & Reich, 2012) on a Zephyr^®^ G3 Compact Liquid Handling Workstation (Caliper Life sciences, USA). Finally, the library was quantified using Qubit dsDNA HS kit (Thermo Fisher Scientific, USA), normalized and pair‐end sequenced on an Illumina MiSeq platform with the v3 Reagent Kit (Illumina), and demultiplexed in MiSeq Reporter (v2.5).

Raw sequences were filtered using a custom pipeline implemented in Geneious V8.1.8 (Biomatters, New Zealand). In short, primer sequences were removed and bases were quality‐trimmed (base quality < 20); subsequently reads were paired, merged (minimum of 10 bp overlap), de novo assembled (1% mismatch allowed) to contigs and blasted against the GenBank COI database for fish and invertebrates. Blast results were quality‐filtered on a low number of reads per sample (<0.1%), low pairwise identity (<98%), and a fragment length outside a 10% range of the expected length (313 bp).

### 2.4 Stable isotope analysis

Stable isotope sampling procedures and quantification followed Matley et al. (2017). Briefly, three tissues (plasma, red blood cells, and muscle) were collected from *Plectropomus* individuals and frozen (−20°C) until processing. Muscle tissue (no skin) was sampled from the dorsal musculature using sterile forceps and scalpel, and blood components were sampled from the 2nd or 3rd gill arch with a sterile needle/syringe. Frozen samples were freeze‐dried for 48 hr and ground into a powder, then samples were lipid‐extracted using a 2:1 chloroform:methanol solvent. Stable isotope values (δ^13^C and δ^15^N) were calculated using a continuous flow isotope ratio mass spectrometer (Finnigan MAT Deltaplus, Thermo—Finnigan) equipped with a Costech Elemental Analyzer (Costech Analytical Technologies). Stable isotope analysis exceeded accepted precision and accuracy standards (Matley et al., 2017).

### 2.5 Data analysis

Unless indicated otherwise, samples from each species were pooled between reefs and dates due to the limited number of individuals sampled. Previous research indicated different color phases of *P. laevis* (bluespot and footballer) have different feeding ecology (Matley et al., 2017); therefore, color phases were analyzed separately. Prey items were grouped by family when visually identified due to low numbers. The family Labridae was subdivided into Scarinae and “all others” because of the different feeding modes exhibited (e.g., parrotfishes are typically herbivores/detritivores, other Labridae are mostly predatory). Dietary indices used to summarize the findings included: percent prey contribution (*N*_*i*_), frequency of occurrence (*O*_*i*_), percent weight (*W*_*i*_), and index of relative importance (*IRI*_*i*_) (following St John, 2001). Prey family composition was plotted after *Plectropomus* were divided into 3 size classes (<450 mm, 450–550 mm, >550 mm) to investigate prey consumption associated with ontogeny/growth.

To investigate whether DNA‐identified stomach contents included a sufficient number of samples to formally analyze, the cumulative number of new prey families within each consecutive stomach sampled (randomly ordered) was plotted for each species using the *specaccum* function within the “vegan” package (Oksanen et al., 2016) in the R environment (R Development Core Team 2014). Samples were considered adequate to characterize the diet if curves approached an asymptote (Ferry & Cailliet, 1996).

Comparison of DNA‐identified stomach contents among species and color phases was facilitated by nonmetric multidimensional scaling (nMDS) based on the presence/absence of prey families using the Bray–Curtis dissimilarity index within the “vegan” package (Oksanen et al., 2016). An analysis of similarity (ANOSIM) tested for significant differences among species and color phases (reefs and sampling periods pooled separately for TSV and OI reefs); a global *R*‐statistic value between −1 and +1 was produced with an associated significance level (*α* = 0.05). More positive *R‐*statistic values indicate between‐group differences, whereas values close to zero indicate random grouping (i.e., within‐ and between‐group dissimilarities are indistinguishable). The degree of DNA‐based dietary overlap between species was tested using the simplified Morisita index and *Plectropomus* species combinations with values above 0.60 were considered to have significantly overlapping diets (Langton, 1982). Differences in DNA stomach contents between TSV reefs (all species and sampling periods combined) and sampling periods (all species and TSV reefs combined) were also tested by ANOSIM as described above. In addition, prey family composition was plotted after *Plectropomus* were divided into 3 size classes (FBT/BST: <450 mm, 450–650 mm, >650 mm; CCT: <450 mm, 450–550 mm, >550 mm; ICT: <300 mm, 300–400 mm, >400 mm) to investigate prey consumption associated with ontogeny/growth.

Although prey abundance/density at each reef was not determined simultaneously with *Plectropomus* sampling, resource selection was estimated using abundance data from previous surveys at four TSV reefs (Helix Reef, Rib Reef, Chicken Reef, and Knife Reef) during March 2014 as part of the Australian Institute of Marine Science (AIMS) Long Term Monitoring Program (Bierwagen et al.—*in press*). Briefly, these surveys incorporated fish counts from 5 m belt transects (1 m for pomacentrids) along five 50 m transects at three sites (i.e., 15 transects). Jacobs’ Electivity Index (*D*; Jacobs, 1974) was calculated using DNA‐based stomach contents of *Plectropomus* at TSV reefs to determine if prey families were specifically selected for independent of their relative abundance within the environment. Jacobs’ *D* was calculated using the equation: *D = r − p*/(*r + p*) *−* (*2rp*), where *r* represents the proportion of a given prey family in the diet and *p* in the environment. The value of *D* varies from 1 (maximum avoidance) to +1 (maximum preference). Index values of 0 indicate that prey species are consumed in proportion to their abundance. Index values were calculated for each survey reef to provide 95% confidence intervals; as a conservative approach, if confidence intervals fell between −0.25 and +0.25, that prey family was considered to be consumed in proportion to its abundance (i.e., neutral selection).

Prey contribution (family‐level) was estimated (at 75% credibility intervals) for each species (and color phase) using Bayesian stable isotope mixing models (adjusted for plasma, RBC, and muscle discrimination factors, respectively—Matley, Fisk et al., 2016) within the “siar” package (Parnell & Jackson, 2013) in R. The diagnostic correlation matrix plot was used to identify prey sources that were similar; when this occurred, model iterations could not distinguish between prey sources resulting in an unknown or biased contribution between sources in the posterior model. To address this, confounding sources were removed or interpreted as a combined source. Priors, based on DNA‐identified stomach contents for each species (described above), were applied with a conservative standard error estimate of 0.078 (roughly equivalent to a 20% confidence interval) to improve prey contribution output (Jackson, Inger, Parnell, & Bearhop, 2011). Prey contribution estimates were similarly calculated using mixing models without prior information. Prey composition overlap comparisons were made between both model outputs (i.e., with and without priors) for each species and color phase, and tissue type (based on the midvalue of 75% credibility intervals) using the simplified Morisita index. Index values above 0.60 indicated that diet composition from mixing model outputs was similar.

The capacity for stable isotope mixing models to accurately characterize prey composition was assessed by comparing the contribution of prey in mixing models with temporally and spatially relevant contributions from DNA‐identified stomach contents. For example, an isotopic sampling of *P. leopardus* conducted in February 2014 at Helix Reef, roughly corresponded to stomach contents of individuals sampled in November 2013 (~90 days). Also, muscle isotopic trends (50% turnover is ~126 days) from February 2014 should incorporate diet from August 2013 sampling (~180 days). It is important to note that a combination of prey signatures gradually become incorporated into consumer tissues over time, and thus, 50% turnover periods used here are an approximate temporal estimate of isotope incorporation.

The trophic level (TL) of each DNA stomach content prey item was determined using estimated values from http://www.fishbase.org (Froese & Pauly, 2016). To test whether different factors (e.g., species, color phase, size, δ^15^N values, and reef) influenced the TL of consumed prey, a general linear model (GLM) was used for each tissue sampled. Parameters were estimated with restricted maximum likelihood and a Gaussian distribution (link: identity). Here, data were subset to the November 2013 (for TSV reefs) and May 2014 (for OI Reef) sampling periods to reduce seasonal isotopic bias and to include all *Plectropomus* species sampled. Model assumptions (e.g., homogeneity of variance and normality) were verified using diagnostic plots and tests were considered significantly different if *p *≤* *0.05.

## 3 RESULTS

Of the 226 stomachs visually examined 100 (44%) contained prey, 31 (23—*P. leopardus*; 5—*P. laevis*; 3—*P. maculatus*) had identifiable prey items (39 different items), which were identified to family or lower (11 of these identified to species). Caesionidae, Labridae, and Pomacentridae were the main prey families and comprised ~80% of identified prey (Table 2; Supporting information Figure S1) and at least one of these families was found in ~71% of individual stomachs with identifiable prey.

**Table 2:** Visual stomach contents including percent prey contribution (%N), frequency of occurrence (%O), percent weight (%W), and index of relative importance (%*IRI*) based on 31 individuals (all *Plectropomus* spp. combined)

	Caesionidae	Labridae	Pomacentridae	Labridae (Scarinae)	Acanthuridae	Serranidae	Siganidae	Crustacea	Gobiidae	Apogonidae
%*N*	26.3	23.7	23.7	5.3	2.6	5.3	2.6	5.3	2.6	2.6
%*O*	32.3	25.8	19.4	6.5	3.2	6.5	3.2	6.5	3.2	3.2
%*W*	45.1	9.0	8.9	17.4	9.1	5.2	7.7	0.6	0.9	0.5
%*IRI*	34.9	16.0	15.9	11.1	5.7	5.1	5.1	2.9	1.7	1.5

*Note*

Stomachs of 226 individuals (–171—*P. leopardus*, –43—*P. laevis*, –12—*P. maculatus*) were examined but only 31 had stomach contents identifiable to family. Prey weight was not adjusted for partial digestion, and consequently, %*W* and %*IRI* are likely underestimated for some families.

**Figure 2:**
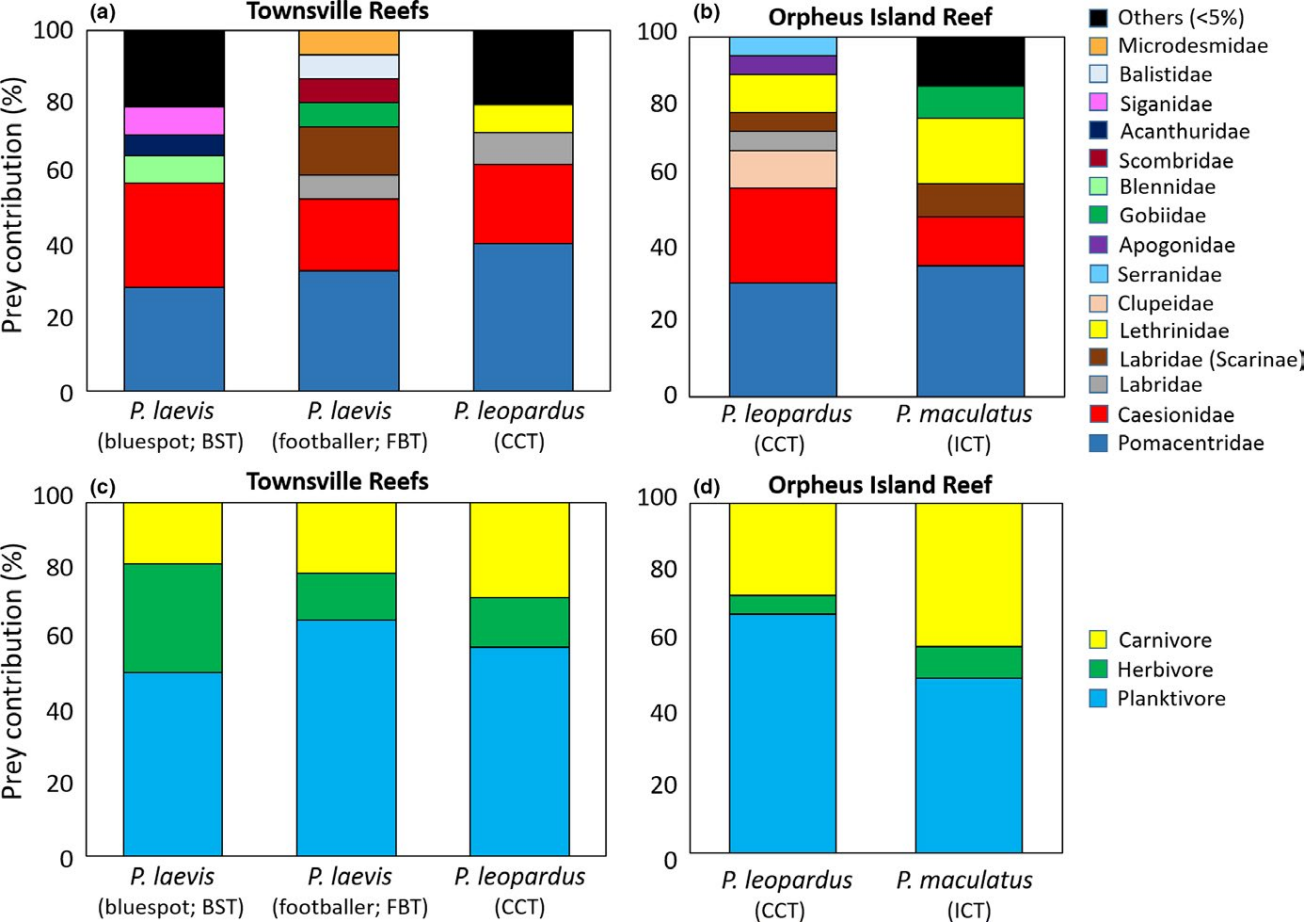
Prey family (a,b) and prey functional mode (c, d) contribution (%*N*) calculated from DNA stomach analysis from 31 *P. leopardus* (CCT), 22 *P. laevis* (bluespot; BST), 10 *P. laevis* (footballer; FBT) captured at Townsville (TSV) reefs (Helix, Coil, and Dip Reefs combined; a, c) and 9 CCT, 9 *P. maculatus* (ICT) captured at Orpheus Island (OI) Reef (b, d). Prey families that consisted of <5% of total prey for each consumer group were combined as “Others”

**Figure 3:**
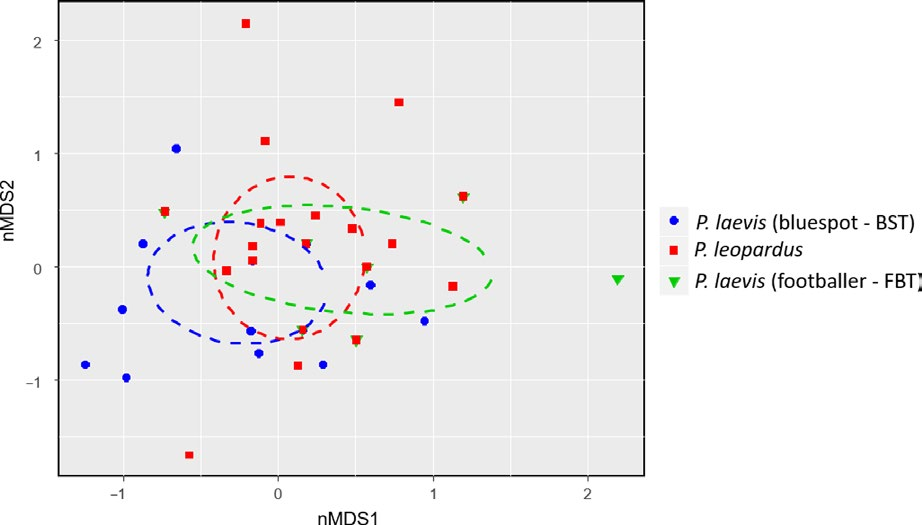
Nonmetric multidimensional scaling (nMDS) plot characterizing DNA stomach analysis relative to prey family of *Plectropomus* spp. at TSV reefs (Helix, Yankee, and Coil Reefs). A two‐dimensional Bray–Curtis dissimilarity index was used resulting in a stress level of 0.08, ANOSIM R‐statistic of 0.05, and *p*‐value of 0.14

Of the stomachs (*n* = 101) sampled for genetic metabarcoding of prey (Table 1), 187 prey items (digestion level 1: *n* = 41, 2: *n* = 33, 3: *n* = 68, 4: *n* = 45) from 81 individuals (40—*P. leopardus*; 32—*P. laevis*; 9—*P. maculatus*) were identified which included 50 species from 20 families (Supporting information Table S1; Supporting information Figure S2). Cumulative prey curves for *P. leopardus* and *P. laevis* approached asymptotes at ~20–25 samples, suggesting sufficient samples to characterize diet (Supporting information Figure S3). The footballer phase of *P. laevis* had <20 samples but was treated separately from the bluespot phase due to previous investigations indicating distinct feeding ecology. Likewise, sample sizes for *P. maculatus* and *P. leopardus* at OI Reef were not adequate, but the main output was included for exploratory purposes. For all species and color phases at TSV reefs and OI Reef, Pomacentridae and Caesionidae comprised >50% of identified prey (Figure 2a,b; Supporting information Figure S4). These mainly included the following species: *Pterocaesio digramma* (Caesionidae), *Neopomacentrus azysron*,* Acanthochromis polyacanthus*, and *Pomacentrus trichrourus* (Pomacentridae) (Supporting information Table S1; Supporting information Figure S2). Remaining prey families varied between species of *Plectropomus*. Planktivores were the most common prey for all species (~50%–70%; Figure 2c,d; Supporting information Figure S5), and herbivores comprised ~10%–15% of prey except in *P. laevis* (bluespot), where it accounted for ~30%. Based on ANOSIM at TSV reefs, prey family differences were not found between *Plectropomus* species/color phases (R‐statistic = 0.050, *p *=* *0.137; Figure 3), reefs (R‐statistic = 0.012, *p *=* *0.292), or sampling periods (R‐statistic* *=* *0.153, *p *=* *0.067). ANOSIM results were similar when prey species (as opposed to families) were compared with *Plectropomus* species/color phases (Supporting information Figure S6) but caution interpreting this output is suggested due to the large number of prey species in relation to *Plectropomus* sample size. Simplified Morisita indices for all TSV species and color phase combinations were >0.80 indicating high dietary overlap.

**Figure 4:**
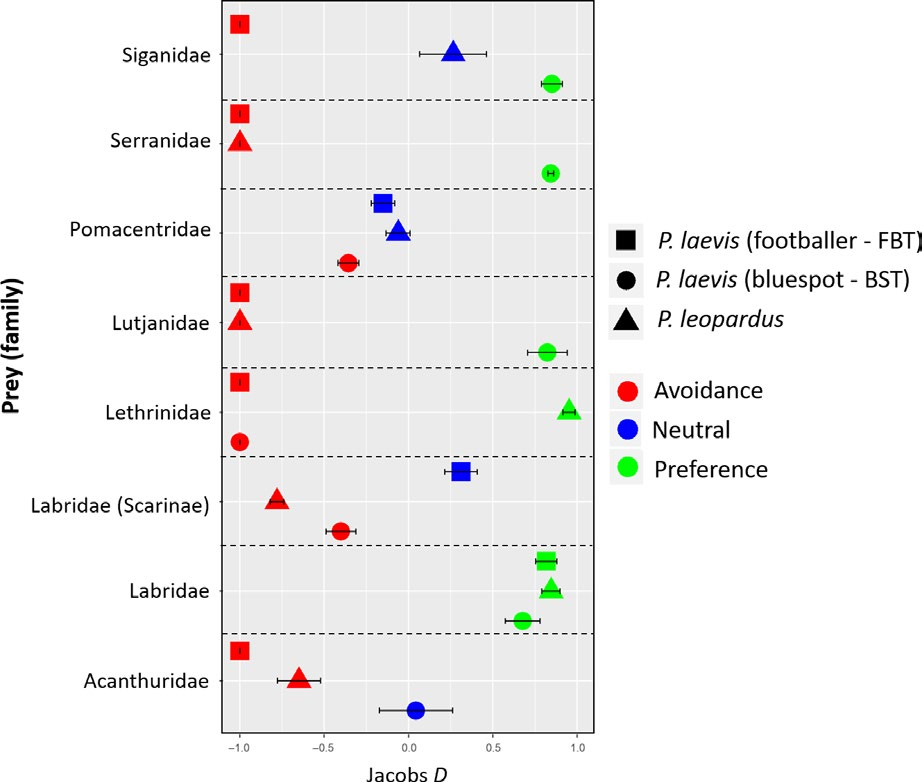
Summary of resource selection as indicated by Jacobs’ Electivity Index (*D*) for *Plectropomus* at TSV reefs. The proportion of prey consumed was calculated using DNA stomach contents and the proportion of prey available was estimated from standardized abundance surveys at four TSV reefs (Helix Reef, Rib Reef, Chicken Reef, and Knife Reef) during 2014 (Bierwagen et al.—*in press*). The value of *D* varies from 1 (maximum avoidance) to +1 (maximum preference). Index values of 0 indicate that prey species are consumed in proportion to their abundance. Confidence intervals that fell between −0.25 and +0.25 were deemed to be consumed in proportion to its abundance (i.e., neutral selection)

Pomacentridae was the most abundant family surveyed during 2014, followed by Labridae (including Scarinae) and Acanthuridae (Bierwagen et al.—*in press*). Prey selection patterns, as determined by Jacobs’ Electivity Index showed selection for Labridae (not including Scarinae) for all *Plectropomus* at TSV reefs (Figure 4). Also, no strong selection or avoidance patterns were readily apparent for Pomacentridae despite its high abundance. Otherwise, the bluespot *P. laevis* selected for Siganidae, Serranidae, and Lutjanidae, whereas *P. leopardus* demonstrated an affinity to Lethrinidae (Figure 4); however, these families contributed only a small portion within the diet of *Plectropomus* (Figure 2). Caesionidae and a few other families found in the stomachs of *Plectropomus* were not included in these abundance surveys and were not included in this analysis.

**Figure 5:**
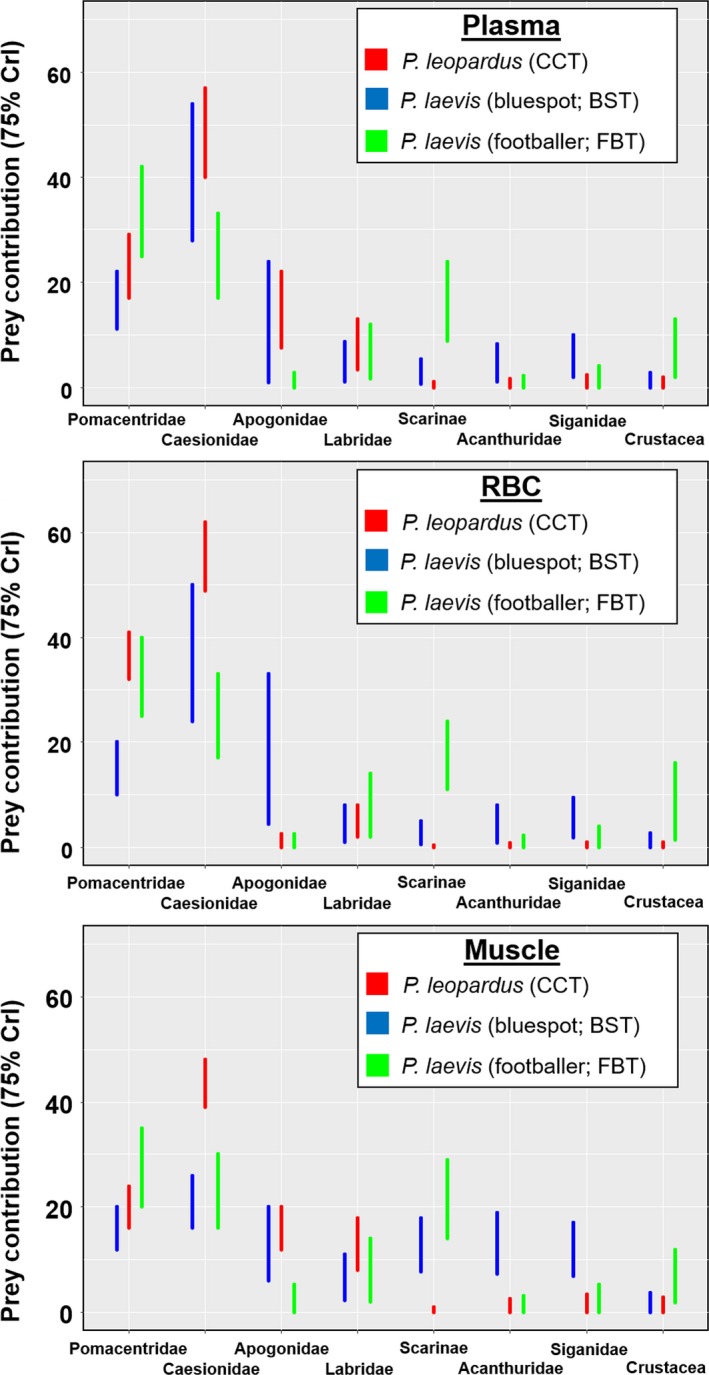
Prey contribution estimates (75% credibility intervals) for *Plectropomus* spp. at TSV reefs (Helix, Yankee, and Coil Reefs combined) based on Bayesian stable isotope mixing models (adjusted for plasma, RBC, and muscle discrimination factors, respectively (Matley, Fisk et al., 2016)) using priors consisting of DNA stomach content analysis for each species. stomach

**Figure 6:**
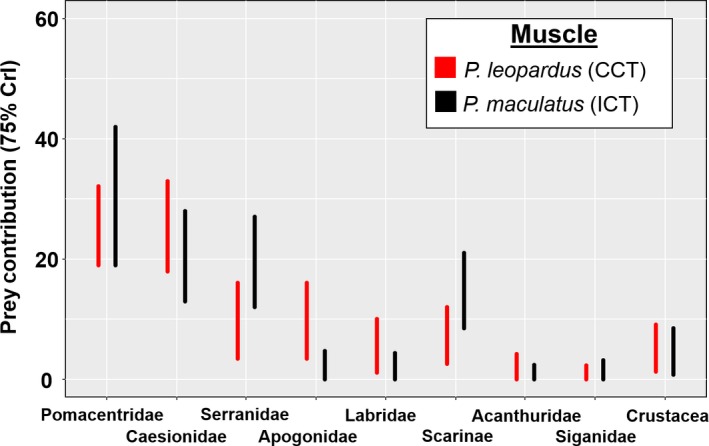
Prey contribution estimates (75% credibility intervals) for *Plectropomus leopardus* (CCT) and *P. maculatus* (ICT) at Orpheus Island Reef based on Bayesian stable isotope mixing models (adjusted for muscle discrimination factors (Matley, Fisk et al., 2016)) using priors consisting of DNA stomach content analysis for each species

Prey contribution based on stable isotope mixing models using DNA‐identified stomach content as prior information mainly consisted of Caesionidae and Pomacentridae for all species and tissues (Figures 5 and 6). At TSV reefs, due to similar isotopic values between these two prey families it was difficult to distinguish their contribution values. Nevertheless, both comprised >60% of diet in *P. leopardus* and *P. laevis* for all tissues sampled (Figure 5). Prey composition overlap between mixing models with and without prior information was significant (>0.60 index) for all tissues of *P. leopardus* (at TSV reefs and OI Reef), *P. maculatus*, and *P. laevis* (footballer); however, prey composition differed for *P. laevis* (bluespot) (all tissues). The main difference between mixing model outputs at TSV reefs was that Pomacentridae and Caesionidae contributed less to the diet when prior information was not included in mixing models, particularly for bluespots which generally showed a greater input of benthic consumers such as Scarinae, Acanthuridae, and Siganidae (Supporting information Figure S7). At OI Reef, Serranidae contributed a larger portion of the diet when prior information was not considered (Supporting information Figure S8).

**Figure 7:**
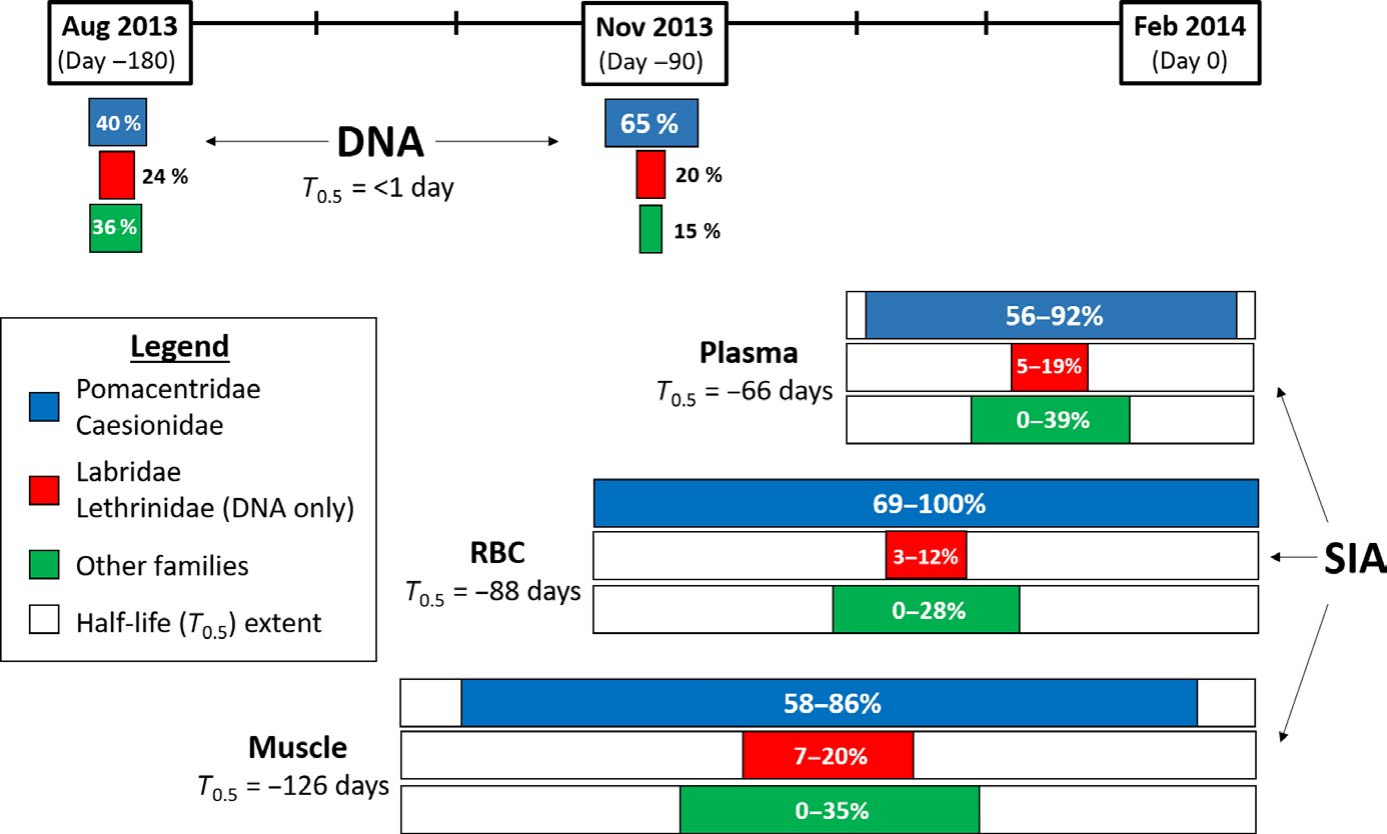
Interpretive representation of stable isotope turnover (i.e., half‐life, T_0.5_) and prey composition estimates from mixing models (using 75% credibility intervals) in different tissues of *Plectropomus leopardus* sampled in February 2014 relative to prey composition based on DNA stomach contents in August and November 2013

Prey composition estimated from spatially and temporally equivalent DNA‐identified stomach contents and stable isotopes was similar (Figure 7). DNA‐identified stomach contents from August and November 2013 at Helix Reef consisted mainly of Pomacentridae and Caesionidae (August: 40% of prey; November: 65%), as well as Labridae and Lethrinidae (August: 24% of prey; November: 20%). Corresponding (i.e., February 2014—Helix Reef) mixing model outputs (with priors) also indicated large contribution of Pomacentridae and Caesionidae (75% credibility intervals of muscle: 58%–86%; RBC: 69%–100%; plasma: 56%–92%), and Labridae (Lethrinidae were not sampled) were also the third most consumed prey (muscle: 7%–20%; RBC: 3%–12%; plasma: 5%–19%).

The GLM testing whether factors such as species, color phase, size, δ^15^N values, and reef affected TL of DNA‐based prey showed that plasma (*F*_1,43_ **=* *13.4, *p *<* *0.001; δ^15^N parameter estimate ± *SE *=* *0.51 ± 0.19) and RBC (*F*_1,42_ **=* *7.5, *p *=* *0.010; δ^15^N parameter estimate ± *SE *=* *0.78 ± 0.21) δ^15^N values of *Plectropomus* at TSV reefs were significant (positive relationship with prey TL). No other factors (including species*size interactions) were significant at TSV reefs or OI Reef (i.e., *p *>* *0.15).

## 4 DISCUSSION

This study demonstrated the utility of multiple sampling techniques to characterize the diet of predatory reef fish. Specifically, DNA stomach analysis provided high prey resolution even when items were degraded. Stable isotopes were useful at interpreting longer‐term dietary patterns, particularly when combined with DNA stomach analysis, demonstrating that when repetitive, long‐term, or lethal sampling is impractical or not possible, stable isotope analysis is a powerful alternative, albeit with less taxonomic resolution. Both methods produced similar patterns among *Plectropomus*, with Caesionidae and Pomacentridae being the main prey.

### 4.1 Methodological implications

Visual stomach content analysis is typically an affordable approach to identify prey but relatively labor‐intensive and limited by biases associated with digestion rates, regurgitation of prey, and empty stomachs (Arrington, Winemiller, Loftus, & Akin, 2002; Vinson & Budy, 2011). These biases can be problematic when interpreting diet for large piscivores because a wide variety of prey is often consumed heterogeneously in space and time (Armstrong & Schindler, 2011). Here, prey could only be visually identified in ~14% of individuals because of empty stomachs (56%) and digested stomach contents (30%). Other studies have had similar limitations for *P. leopardus* (Kingsford, 1992; St John, 1999). Unless sampling can be conducted on many individuals (e.g., >20–25 individuals with identifiable prey per sampling category), visual stomach content analysis alone may be impractical for fishes with conservation concerns such as *Plectropomus*.

The use of molecular approaches, especially next‐generation sequencing (NGS) barcoding, to identify prey of fishes is relatively new. However, these methods are increasingly utilized to identify prey and explore ecological implications of diet (e.g., Leray et al., 2013, 2015). Here the molecular approach identified prey in ~80% of individuals, including stomachs that were qualified as empty by visual analysis. Likewise, Barnett, Redd, Frusher, Stevens, and Semmens (2010) doubled the number of identifiable prey compared to morphological analysis in broadnose sevengill sharks *(Notorynchus cepedianus*). Further, more prey items were detected in each stomach compared to visual methods. For example, ~26% of stomachs with identifiable prey contained two or more items based on visual stomach contents (this study; St John, 1999), but ~69% of stomachs had two or more items based on DNA identification. The main drawback associated with the DNA approach is cost (e.g., ~50AUD per individual) and the need for prior validation; however, once optimized, more samples can be analyzed simultaneously at relatively lower costs. In addition, region‐specific genetic markers are needed to broaden available databases and avoid taxonomic uncertainty, especially for rare species. Nevertheless, the ability to successfully identify prey after many hours of digestion (<16 hr; Carreon‐Martinez et al., 2011) provides greater scope to characterize short‐term dietary patterns compared to conventional methods.

Stable isotopes are now readily used as an alternative or supplement to stomach content analysis. The specific advantage is that broad‐scale feeding patterns reflecting habitat and prey sources can be inferred at multiple temporal scales (Newsome et al., 2010). In addition, lethal sampling is not necessary and there is no bias associated with digestion rates or empty stomachs (Colborne, Clapp, Longstaffe, & Neff, 2015). A major limitation with isotope analysis is to obtain a comprehensive view of diet, isotopically distinct prey species typically need to be sampled, which can be difficult and inflate costs (~15‐30AUD per sample). Here mixing models were difficult to statistically compare with stomach content results because of the greater temporal scale associated with tissue‐specific isotopic assimilation. Thus, this study is unable to specifically compare prey composition among the different approaches because diet manipulation and standardization were not completed. In addition, not all prey types detected in the stomachs were sampled for stable isotope values. Nevertheless, based on the corresponding temporal proxies of diet between February 2014 muscle tissue and August 2013 DNA‐identified stomach contents, and between February 2014 blood components and November 2013 DNA‐identified stomach contents, the dietary output from mixing models was typically within estimated margins for DNA‐identified stomach contents for *P. leopardus* at Helix Reef. Admittedly, stable isotope mixing models incorporated stomach content data, but conservative margins of error (i.e., 20% confidence interval) were used to not guide the models too strongly and mixing models without prior information still identified the main prey groups.

Results of this analysis highlight the value of using multiple complementary approaches. The visual analysis provided a baseline understanding of diet, but lacked the detail and resolution provided by the molecular approach. These results, in combination with long‐term information supplied by isotope analysis, provide a more comprehensive understanding of feeding patterns. The inclusion of prior knowledge (i.e., stomach content data) into Bayesian mixing models has the ability to improve precision in estimating diet composition at monthly temporal scales (Chiaradia et al., 2014; Franco‐Trecu et al., 2013). It was particularly useful identifying prey composition in *P. laevis* (bluespot), which appeared to underestimate the contribution of Pomacentridae and Caesionidae when prior information was not incorporated. If possible, future studies should validate both techniques at multiple and mutually relevant time‐scales to track seasonal dietary changes that may occur among species (not apparent in this study).

### 4.2 Ecological implications

This study demonstrated that planktivorous Pomacentridae and Caesionidae are important components of *Plectropomus* diet at short‐ and long‐term temporal scales. This has been previously demonstrated for *P. leopardus* based on stomach content results (Kingsford, 1992; St John, 1999). *Plectropomus leopardus* are often considered opportunistic generalists consuming prey relative to their abundance within coral reef systems; however, this has not specifically been tested. A more comprehensive sampling regime is needed to fully understand resource selection patterns for *Plectropomus*; however, the preliminary investigation of prey selection in this study supports this conclusion for *P. leopardus* and *P. laevis*, particularly considering that Pomacentridae were dominant in the diet. Although Caesionidae were not sampled in the abundance surveys utilized in this study, video‐recorded surveys with similar sampling design have shown that Pomacentridae and Caesionidae are overwhelmingly the most abundant families on the TSV reefs (Stacy Bierwagen, pers. comm.). Therefore, *Plectropomus* appear to follow generalist and opportunistic prey selection, at least for a large component of their diet. Notwithstanding relative abundance of Pomacentridae and Caesionidae, planktivorous species in these families may be more vulnerable to predation when foraging above reef structure because *Plectropomus* are ambush predators (St John, 2001).

The similarity of prey selection among *Plectropomus* species has ecological implications within coral reef ecosystems. Shared resource‐use among *Plectropomus* could lead to competitive interactions or altered community composition (Boström‐Einarsson, Bonin, Munday, & Jones, 2014; Papastamatiou, Wetherbee, Lowe, & Crow, 2006). This issue will likely be amplified under predicted climate change scenarios if metabolic demands of large predators are not met due to prey availability (Johansen et al., 2015; Pörtner & Peck, 2010) and habitat degradation alters community composition (Jones, McCormick, Srinivasan, & Eagle, 2004; Wen, Bonin, Harrison, Williamson, & Jones, 2016). Although *Plectropomus* are likely capable of adapting to changing resource pools (Graham et al., 2007; Johansen et al., 2015), planktonic food sources are important. Indeed, the four main prey species (*P. digramma, N. azysron*,* A. polyacanthus*, and *P. trichrourus*) are predominantly planktivorous (Froese & Pauly, 2016). Changes in primary production and plankton‐based trophodynamics (e.g., Doney, Fabry, Feely, & Kleypas, 2009) will likely have a strong effect on how mesopredators such as *Plectropomus*, select and partition prey (Audzijonyte, Kuparinen, Gorton, & Fulton, 2013; Hempson et al., 2017).

Despite major prey items being similar among species and color phases, their contribution, and that of lesser prey differed. For example, more planktonic prey (Clupeidae, Caesionidae) were detected in *P. leopardus* compared to *P. maculatus*, which consumed more benthic/midwater consumers (Gobiidae, Lethrinidae), at OI Reef. This difference may be a result of vertical segregation (Matley, Heupel et al., 2016), but a larger sample size is needed to confirm these results. Bluespot *P. laevis* appeared to select predatory consumers such as Serranidae, Lutjanidae; however, low abundances of these families in surveys may have overinflated the few found in stomachs. Benthic herbivores (Acanthuridae, Blenniidae, Siganidae) were ~15%–20% more abundant in bluespot DNA stomach contents compared to other species and color phases. Differences in benthic carbon sources between *P. leopardus* and *P. laevis* (bluespot) were also found based on isotopic niche breadth, which showed limited overlap (0%–21%) for plasma, RBC, and muscle tissue (Matley et al., 2017). Within‐reef isotopic composition of prey likely varies based on physical and biological processes associated with habitat or location on the reef (e.g., depth or proximity to ocean floor; Wyatt, Waite, & Humphries, 2012). Therefore, the same prey species may have different isotope values depending on foraging habitat. This may help explain why the stomach contents of *P. leopardus* and *P. laevis* were not markedly different, as opposed to isotopic niche breadth, as both species exhibited different home ranges and movement patterns (i.e., different foraging modes; Matley, Tobin, Ledee, Heupel, & Simpfendorfer, 2016). Different feeding modes within prey families may also drive isotopic differences. Further explorations of within‐reef isotopic variation are needed to assess the extent to which habitat and primary production influence values at a local scale.

Temporal changes in feeding patterns within and between species and color phases were identified. In mixing models, benthic prey groups contributed more to *P. laevis* (bluespot) muscle tissue compared to plasma and RBCs, indicating that over a longer timescale, benthic, and midwater prey were consumed by bluespots. In contrast, tissue‐specific differences in prey composition (based on mixing models) were not evident within *P. laevis* (footballer) or *P. leopardus*. Prey abundance surveys incorporated in this study were limited to one period (March 2014) outside of DNA sampling, and therefore, seasonal fluctuations in recruitment could affect prey selectivity; however, past research has found that the diet of *P. leopardus* does not change seasonally (St John, 2001). Findings in this study align with this for *P. leopardus*, as well as *P. laevis* (footballer)*,* which is hypothesized to have an intermediate feeding ecology between *P. laevis* (bluespot) and *P. leopardus* (Matley et al., 2017).

Consumers typically select prey that optimize energetic gains such as larger prey (offset by foraging costs; Pyke et al. 1977). However, the size of consumed prey is often limited by consumer size due to limitations such as gape size (Mittelbach and Persson 1998). In this study, there was no strong evidence of *Plectropomus* size affecting prey size as demonstrated by the GLM with prey TL as the response variable; however, within‐species differences in size could not be tested. Likewise, when *Plectropomus* were divided into different size classes, there was no evident separation in major prey groups, but more equitable sample sizes would aid interpreting this output. These findings are not surprising because ontogenetic shifts in prey selection mainly occur prior to maturity (St John, 1999; Wen, Almany, Williamson, Pratchett, & Jones, 2012).

## 5 CONCLUSION

This study showed DNA stomach analysis was a more comprehensive tool to characterize diet compared to visual stomach analysis by increasing resolution of prey identification and detecting greater taxonomical diversity. The use of stable isotopes provided dietary estimates over longer periods of time and quantification of prey via Bayesian mixing models matched well with temporally congruent samples after incorporating stomach content information. *Plectropomus* δ^15^N values in plasma and RBCs reflected the TL of prey; however, muscle δ^15^N values did not, highlighting the limitations of stomach contents to characterize diet over longer periods. Thus, interpretation of muscle‐derived mixing models should be treated cautiously because greater uncertainty in prey items exists. Still, similarities between temporally relevant dietary output were identified, suggesting prey isotope values, discrimination factors, and prior stomach content information were suitably applied. Thus, unless repeat sampling of sufficient sample size is possible to acquire prey via stomach contents, multi‐tissue stable isotope investigations provide a valuable alternative, particularly if combined with well‐informed (and temporally relevant) stomach content analysis. Small sample sizes, particularly at OI Reef and for footballer *P. laevis*, hindered the reliability of comparisons among species and extent of conclusions that could be made in this study. However, like in most studies, sample sizes were constrained by several factors such as processing costs and legal catch‐limits. Broad‐scale prey selection patterns among *Plectropomus* were evident despite sample sizes, with Caesionidae and Pomacentridae as the main contributors for all species. The implication of a shared diet among *Plectropomus* is relevant to resource managers because changing environmental conditions will likely have a strong effect on prey availability and resource partitioning among predators. Furthermore, based on prey composition of *Plectropomus*, plankton‐based resources play a key role in structuring energetic pathways of predatory reef fish.

## CONFLICT OF INTEREST

None declared.

## AUTHORS’ CONTRIBUTIONS

JK Matley and AJ Tobin collected most samples; JK Matley and AT Fisk processed and analyzed isotope samples; JK Matley, GE Maes, F Devloo‐Delva, R Huerlimann, and G Chua processed and analyzed genetic samples; and all authors were involved in the concept and design of the project, data analysis, and contributed to writing/revisions.

## DATA ACCESSIBILITY STATEMENT

Supporting data have been uploaded to Dryad (doi: 10.5061/dryad.4v80379).

## Supporting information


** **
