# Supplementary material for: Integrating complementary methods to improve diet analysis in fishery‐targeted species
Source: Ecol Evol. 2018 Aug 29;8(18):9503–15. doi: 10.1002/ece3.4456 (PMC6194329; doi:10.1002/ece3.4456)
Supplement: Supplementary file 1 [file ECE3-8-9503-s001.docx]

Supplementary Table S1: Summary of prey identified by DNA gut contents of *Plectropomus* spp. Multiple items detected in the same individual are denoted by the same ‘Host ID’ number.

| **Host ID** | **Host species** | **Date** | **Reef** | **Prey family** | **Prey species** | **Common Name** |
| --- | --- | --- | --- | --- | --- | --- |
| 364375 | *Plectropomus laevis* (bluespot) | Nov-2013 | Dip | Caesionidae | *Caesio caerulaurea* | blue and gold fusilier |
| 364375 | *Plectropomus laevis* (bluespot) | Nov-2013 | Dip | Caesionidae | *Pterocaesio digramma* | double-lined fusilier |
| 345516 | *Plectropomus laevis* (bluespot) | Nov-2013 | Dip | Blenniidae | *Cirripectes stigmaticus* | red-streaked blenny |
| 345516 | *Plectropomus laevis* (bluespot) | Nov-2013 | Dip | Pomacentridae | *Pomacentrus bankanensis* | speckled damselfish |
| 364365 | *Plectropomus laevis* (bluespot) | Nov-2013 | Dip | Pomacentridae | *Acanthochromis polyacanthus* | spiny chromis |
| 364365 | *Plectropomus laevis* (bluespot) | Nov-2013 | Dip | Nemipteridae | *Scolopsis monogramma* | monogrammed monocle bream |
| 345518 | *Plectropomus laevis* (bluespot) | Nov-2013 | Dip | Caesionidae | *Pterocaesio digramma* | double-lined fusilier |
| 345519 | *Plectropomus laevis* (bluespot) | Nov-2013 | Dip | Blenniidae | *Cirripectes stigmaticus* | red-streaked blenny |
| 345519 | *Plectropomus laevis* (bluespot) | Nov-2013 | Dip | Pomacentridae | *Acanthochromis polyacanthus* | spiny chromis |
| 345520 | *Plectropomus laevis* (bluespot) | Nov-2013 | Dip | Pomacentridae | *Acanthochromis polyacanthus* | spiny chromis |
| 345520 | *Plectropomus laevis* (bluespot) | Nov-2013 | Dip | Caesionidae | *Pterocaesio digramma* | double-lined fusilier |
| 364366 | *Plectropomus laevis* (bluespot) | Nov-2013 | Dip | Labridae | *Leptoscarus vaigiensis* | marbled parrotfish |
| 364366 | *Plectropomus laevis* (bluespot) | Nov-2013 | Dip | Pomacentridae | *Acanthochromis polyacanthus* | spiny chromis |
| 364366 | *Plectropomus laevis* (bluespot) | Nov-2013 | Dip | Caesionidae | *Pterocaesio digramma* | double-lined fusilier |
| 364368 | *Plectropomus laevis* (bluespot) | Nov-2013 | Dip | Pomacentridae | *Stegastes nigricans* | dusky farmerfish |
| 364368 | *Plectropomus laevis* (bluespot) | Nov-2013 | Dip | Labridae | *Cirrhilabrus punctatus* | dotted wrasse |
| 364368 | *Plectropomus laevis* (bluespot) | Nov-2013 | Dip | Caesionidae | *Pterocaesio digramma* | double-lined fusilier |
| 345515 | *Plectropomus laevis* (bluespot) | Nov-2013 | Dip | Blenniidae | *Cirripectes stigmaticus* | red-streaked blenny |
| 345515 | *Plectropomus laevis* (bluespot) | Nov-2013 | Dip | Caesionidae | *Pterocaesio digramma* | double-lined fusilier |
| 364378 | *Plectropomus laevis* (bluespot) | Nov-2013 | Dip | Caesionidae | *Pterocaesio digramma* | double-lined fusilier |
| 364378 | *Plectropomus laevis* (bluespot) | Nov-2013 | Dip | Lutjanidae | *Lutjanus argentimaculatus* | magrove red snapper |
| 364378 | *Plectropomus laevis* (bluespot) | Nov-2013 | Dip | Serranidae | *Epinephelus merra* | honeycomb grouper |
| 345523 | *Plectropomus laevis* (bluespot) | Nov-2013 | Dip | Acanthuridae | *Acanthurus nigrofuscus* | brown surgeonfish |
| 345523 | *Plectropomus laevis* (bluespot) | Nov-2013 | Dip | Siganidae | *Siganus spinus* | little spinefoot |
| 345523 | *Plectropomus laevis* (bluespot) | Nov-2013 | Dip | Caesionidae | *Pterocaesio digramma* | double-lined fusilier |
| 345514 | *Plectropomus laevis* (bluespot) | Nov-2013 | Dip | Acanthuridae | *Ctenochaetus striatus* | striated surgeonfish |
| 345514 | *Plectropomus laevis* (bluespot) | Nov-2013 | Dip | Siganidae | *Siganus spinus* | little spinefoot |
| 345517 | *Plectropomus laevis* (bluespot) | Nov-2013 | Dip | Holocentridae | *Neoniphon sp.* | Squirrel/soldier fish |
| 345517 | *Plectropomus laevis* (bluespot) | Nov-2013 | Dip | Scombridae | *Scomberomorus semifasciatus* | broadbarred king mackerel |
| 362533 | *Plectropomus laevis* (bluespot) | Nov-2013 | Glow | Acanthuridae | *Ctenochaetus striatus* | striated surgeonfish |
| 362533 | *Plectropomus laevis* (bluespot) | Nov-2013 | Glow | Pomacentridae | *Acanthochromis polyacanthus* | spiny chromis |
| 362533 | *Plectropomus laevis* (bluespot) | Nov-2013 | Glow | Labridae | *Cirrhilabrus punctatus* | dotted wrasse |
| 362524 | *Plectropomus laevis* (bluespot) | Nov-2013 | Glow | Blenniidae | *Cirripectes stigmaticus* | red-streaked blenny |
| 362524 | *Plectropomus laevis* (bluespot) | Nov-2013 | Glow | Caesionidae | *Pterocaesio digramma* | double-lined fusilier |
| 362531 | *Plectropomus laevis* (bluespot) | Nov-2013 | Glow | Pomacentridae | *Pomacentrus trichrourus* | paletail damsel |
| 362531 | *Plectropomus laevis* (bluespot) | Nov-2013 | Glow | Serranidae | *Cephalopholis boenak* | chocolate hind |
| 362531 | *Plectropomus laevis* (bluespot) | Nov-2013 | Glow | Scombridae | *Scomberomorus semifasciatus* | broadbarred king mackerel |
| 346156 | *Plectropomus laevis* (bluespot) | Nov-2013 | Glow | Pomacentridae | *Pomacentrus trichrourus* | paletail damsel |
| 346156 | *Plectropomus laevis* (bluespot) | Nov-2013 | Glow | Caesionidae | *Pterocaesio digramma* | double-lined fusilier |
| 346151 | *Plectropomus laevis* (bluespot) | Nov-2013 | Glow | Pomacentridae | *Pomacentrus amboinensis* | Ambon damsel |
| 346151 | *Plectropomus laevis* (bluespot) | Nov-2013 | Glow | Pomacentridae | *Pomacentrus trichrourus* | paletail damsel |
| 362520 | *Plectropomus laevis* (bluespot) | Nov-2013 | Glow | Pomacentridae | *Acanthochromis polyacanthus* | spiny chromis |
| 362520 | *Plectropomus laevis* (bluespot) | Nov-2013 | Glow | Caesionidae | *Caesio caerulaurea* | blue and gold fusilier |
| 362520 | *Plectropomus laevis* (bluespot) | Nov-2013 | Glow | Caesionidae | *Pterocaesio digramma* | double-lined fusilier |
| 362520 | *Plectropomus laevis* (bluespot) | Nov-2013 | Glow | Pomacentridae | *Neopomacentrus azysron* | yellow-tail demoiselle |
| 346152 | *Plectropomus laevis* (bluespot) | Nov-2013 | Glow | Labridae | *Scarus sp.* | parrotfish |
| 346152 | *Plectropomus laevis* (bluespot) | Nov-2013 | Glow | Pomacentridae | *Pomacentrus trichrourus* | paletail damsel |
| 346152 | *Plectropomus laevis* (bluespot) | Nov-2013 | Glow | Caesionidae | *Caesio caerulaurea* | blue and gold fusilier |
| 346152 | *Plectropomus laevis* (bluespot) | Nov-2013 | Glow | Caesionidae | *Pterocaesio tile* | dark-banded fusilier |
| 346157 | *Plectropomus laevis* (bluespot) | Nov-2013 | Glow | Siganidae | *Siganus spinus* | little spinefoot |
| 346157 | *Plectropomus laevis* (bluespot) | Nov-2013 | Glow | Pomacentridae | *Neopomacentrus azysron* | yellow-tail demoiselle |
| 364397 | *Plectropomus laevis* (bluespot) | Nov-2013 | Helix | Siganidae | *Siganus spinus* | little spinefoot |
| 364387 | *Plectropomus laevis* (footballer) | Nov-2013 | Dip | Balistidae | *Sufflamen fraenatum* | masked triggerfish |
| 364376 | *Plectropomus laevis* (footballer) | Nov-2013 | Dip | Pomacentridae | *Acanthochromis polyacanthus* | spiny chromis |
| 364376 | *Plectropomus laevis* (footballer) | Nov-2013 | Dip | Labridae | *Thalassoma amblycephalum* | bluehead wrasse |
| 364376 | *Plectropomus laevis* (footballer) | Nov-2013 | Dip | Pomacentridae | *Neopomacentrus azysron* | yellow-tail demoiselle |
| 364380 | *Plectropomus laevis* (footballer) | Nov-2013 | Dip | Labridae | *Leptoscarus vaigiensis* | marbled parrotfish |
| 364380 | *Plectropomus laevis* (footballer) | Nov-2013 | Dip | Pomacentridae | *Acanthochromis polyacanthus* | spiny chromis |
| 364380 | *Plectropomus laevis* (footballer) | Nov-2013 | Dip | Caesionidae | *Pterocaesio digramma* | double-lined fusilier |
| 362517 | *Plectropomus laevis* (footballer) | Nov-2013 | Glow | Caesionidae | *Pterocaesio digramma* | double-lined fusilier |
| 362523 | *Plectropomus laevis* (footballer) | Nov-2013 | Glow | Pomacentridae | *Acanthochromis polyacanthus* | spiny chromis |
| 362523 | *Plectropomus laevis* (footballer) | Nov-2013 | Glow | Gobiidae | *Valenciennea longipinnis* | long-finned goby |
| 362529 | *Plectropomus laevis* (footballer) | Nov-2013 | Glow | Scombridae | *Scomberomorus semifasciatus* | broadbarred king mackerel |
| 346154 | *Plectropomus laevis* (footballer) | Nov-2013 | Glow | Labridae | *Leptoscarus vaigiensis* | marbled parrotfish |
| 346155 | *Plectropomus laevis* (footballer) | Nov-2013 | Glow | Caesionidae | *Pterocaesio digramma* | double-lined fusilier |
| 10176 | *Plectropomus laevis* (footballer) | Feb-2014 | Helix | Pomacentridae | *Acanthochromis polyacanthus* | spiny chromis |
| 364351 | *Plectropomus laevis* (footballer) | Nov-2013 | Helix | Microdesmidae | *Ptereleotris evides* | blackfin dartfish |
| 364382 | *Plectropomus leopardus* | Nov-2013 | Dip | Pomacentridae | *Chromis ternatensis* | ternate chromis |
| 364374 | *Plectropomus leopardus* | Nov-2013 | Dip | Pomacentridae | *Acanthochromis polyacanthus* | spiny chromis |
| 364374 | *Plectropomus leopardus* | Nov-2013 | Dip | Gobiidae | *Valenciennea longipinnis* | long-finned goby |
| 345511 | *Plectropomus leopardus* | Nov-2013 | Dip | Caesionidae | *Pterocaesio digramma* | double-lined fusilier |
| 364388 | *Plectropomus leopardus* | Nov-2013 | Dip | Myctophidae | *Diaphus danae* | Dana lanternfish |
| 364388 | *Plectropomus leopardus* | Nov-2013 | Dip | Caesionidae | *Pterocaesio tile* | dark-banded fusilier |
| 364388 | *Plectropomus leopardus* | Nov-2013 | Dip | Pomacentridae | *Lepidozygus tapeinosoma* | fusilier damselfish |
| 345522 | *Plectropomus leopardus* | Nov-2013 | Dip | Pomacentridae | *Pomacentrus trichrourus* | paletail damsel |
| 345522 | *Plectropomus leopardus* | Nov-2013 | Dip | Holocentridae | *Neoniphon sp.* | Squirrel/soldier fish |
| 345524 | *Plectropomus leopardus* | Nov-2013 | Dip | Pomacentridae | *Stegastes fasciolatus* | Pacific gregory |
| 345524 | *Plectropomus leopardus* | Nov-2013 | Dip | Pomacentridae | *Pomacentrus trichrourus* | paletail damsel |
| 345524 | *Plectropomus leopardus* | Nov-2013 | Dip | Caesionidae | *Pterocaesio digramma* | double-lined fusilier |
| 345525 | *Plectropomus leopardus* | Nov-2013 | Glow | Pomacentridae | *Acanthochromis polyacanthus* | spiny chromis |
| 345525 | *Plectropomus leopardus* | Nov-2013 | Glow | Pomacentridae | *Pomacentrus trichrourus* | paletail damsel |
| 362515 | *Plectropomus leopardus* | Nov-2013 | Glow | Caesionidae | *Pterocaesio digramma* | double-lined fusilier |
| 362519 | *Plectropomus leopardus* | Nov-2013 | Glow | Labridae | *Cirrhilabrus punctatus* | dotted wrasse |
| 362519 | *Plectropomus leopardus* | Nov-2013 | Glow | Labridae | *Hemigymnus melapterus* | thicklip wrasse |
| 362521 | *Plectropomus leopardus* | Nov-2013 | Glow | Pomacentridae | *Pomacentrus bankanensis* | speckled damselfish |
| 362521 | *Plectropomus leopardus* | Nov-2013 | Glow | Caesionidae | *Pterocaesio digramma* | double-lined fusilier |
| 345527 | *Plectropomus leopardus* | Nov-2013 | Glow | Pomacentridae | *Acanthochromis polyacanthus* | spiny chromis |
| 345527 | *Plectropomus leopardus* | Nov-2013 | Glow | Pomacentridae | *Pomacentrus trichrourus* | paletail damsel |
| 345527 | *Plectropomus leopardus* | Nov-2013 | Glow | Labridae | *Cirrhilabrus punctatus* | dotted wrasse |
| 345527 | *Plectropomus leopardus* | Nov-2013 | Glow | Caesionidae | *Pterocaesio digramma* | double-lined fusilier |
| 345526 | *Plectropomus leopardus* | Nov-2013 | Glow | Pomacentridae | *Pomacentrus bankanensis* | speckled damselfish |
| 345526 | *Plectropomus leopardus* | Nov-2013 | Glow | Pomacentridae | *Acanthochromis polyacanthus* | spiny chromis |
| 345531 | *Plectropomus leopardus* | Nov-2013 | Glow | Pomacentridae | *Neopomacentrus azysron* | yellow-tail demoiselle |
| 345528 | *Plectropomus leopardus* | Nov-2013 | Glow | Blenniidae | *Cirripectes filamentosus* | filamentous blenny |
| 345528 | *Plectropomus leopardus* | Nov-2013 | Glow | Caesionidae | *Pterocaesio digramma* | double-lined fusilier |
| 345528 | *Plectropomus leopardus* | Nov-2013 | Glow | Pomacentridae | *Neopomacentrus azysron* | yellow-tail demoiselle |
| 362525 | *Plectropomus leopardus* | Nov-2013 | Glow | Caesionidae | *Pterocaesio digramma* | double-lined fusilier |
| 362525 | *Plectropomus leopardus* | Nov-2013 | Glow | Pomacentridae | *Neopomacentrus azysron* | yellow-tail demoiselle |
| 345545 | *Plectropomus leopardus* | Aug-2013 | Helix | Pomacentridae | *Pomacentrus bankanensis* | speckled damselfish |
| 345505 | *Plectropomus leopardus* | Aug-2013 | Helix | Acanthuridae | *Ctenochaetus sp.* | surgeonfish |
| 345550 | *Plectropomus leopardus* | Aug-2013 | Helix | Lethrinidae | *Lethrinus miniatus* | trumpet emperor |
| 345546 | *Plectropomus leopardus* | Aug-2013 | Helix | Pomacentridae | *Pomacentrus trichrourus* | paletail damsel |
| 345546 | *Plectropomus leopardus* | Aug-2013 | Helix | Pomacentridae | *Neopomacentrus azysron* | yellow-tail demoiselle |
| 345546 | *Plectropomus leopardus* | Aug-2013 | Helix | Gobiidae | *Valenciennea strigata* | blueband goby |
| 345546 | *Plectropomus leopardus* | Aug-2013 | Helix | Lethrinidae | *Lethrinus miniatus* | trumpet emperor |
| 345546 | *Plectropomus leopardus* | Aug-2013 | Helix | Scombridae | *Scomberomorus semifasciatus* | broadbarred king mackerel |
| 345547 | *Plectropomus leopardus* | Aug-2013 | Helix | Labridae | *Leptoscarus vaigiensis* | marbled parrotfish |
| 345547 | *Plectropomus leopardus* | Aug-2013 | Helix | Caesionidae | *Pterocaesio digramma* | double-lined fusilier |
| 345547 | *Plectropomus leopardus* | Aug-2013 | Helix | Pomacentridae | *Neopomacentrus azysron* | yellow-tail demoiselle |
| 345547 | *Plectropomus leopardus* | Aug-2013 | Helix | Gobiidae | *Valenciennea strigata* | blueband goby |
| 345547 | *Plectropomus leopardus* | Aug-2013 | Helix | Scombridae | *Scomberomorus semifasciatus* | broadbarred king mackerel |
| 345469 | *Plectropomus leopardus* | Aug-2013 | Helix | Blenniidae | *Cirripectes stigmaticus* | red-streaked blenny |
| 345469 | *Plectropomus leopardus* | Aug-2013 | Helix | Caesionidae | *Pterocaesio digramma* | double-lined fusilier |
| 345469 | *Plectropomus leopardus* | Aug-2013 | Helix | Pomacentridae | *Neopomacentrus azysron* | yellow-tail demoiselle |
| 8351 | *Plectropomus leopardus* | Aug-2013 | Helix | Siganidae | *Siganus spinus* | little spinefoot |
| 8351 | *Plectropomus leopardus* | Aug-2013 | Helix | Pomacentridae | *Pomacentrus trichrourus* | paletail damsel |
| 8351 | *Plectropomus leopardus* | Aug-2013 | Helix | Labridae | *Cirrhilabrus punctatus* | dotted wrasse |
| 8351 | *Plectropomus leopardus* | Aug-2013 | Helix | Caesionidae | *Pterocaesio digramma* | double-lined fusilier |
| 8351 | *Plectropomus leopardus* | Aug-2013 | Helix | Pomacentridae | *Neopomacentrus azysron* | yellow-tail demoiselle |
| 8351 | *Plectropomus leopardus* | Aug-2013 | Helix | Labridae | *Thalassoma hardwicke* | sixbar wrasse |
| 8351 | *Plectropomus leopardus* | Aug-2013 | Helix | Lethrinidae | *Lethrinus miniatus* | trumpet emperor |
| 345543 | *Plectropomus leopardus* | Aug-2013 | Helix | Clupeidae | *Sardinella longiceps* | Indian oil sardine |
| 366526 | *Plectropomus leopardus* | Feb-2014 | Helix | Caesionidae | *Pterocaesio digramma* | double-lined fusilier |
| 10203 | *Plectropomus leopardus* | Jul-2014 | Helix | Labridae | *Cirrhilabrus punctatus* | dotted wrasse |
| 10203 | *Plectropomus leopardus* | Jul-2014 | Helix | Pomacentridae | *Neopomacentrus azysron* | yellow-tail demoiselle |
| 364391 | *Plectropomus leopardus* | Nov-2013 | Helix | Clupeidae | *Sardinella longiceps* | Indian oil sardine |
| 364391 | *Plectropomus leopardus* | Nov-2013 | Helix | Pomacentridae | *Pomacentrus trichrourus* | paletail damsel |
| 364391 | *Plectropomus leopardus* | Nov-2013 | Helix | Pomacentridae | *Amblyglyphidodon leucogaster* | yellowbelly damselfish |
| 364391 | *Plectropomus leopardus* | Nov-2013 | Helix | Caesionidae | *Caesio caerulaurea* | blue and gold fusilier |
| 364391 | *Plectropomus leopardus* | Nov-2013 | Helix | Caesionidae | *Caesio teres* | yellow and blueback fusilier |
| 364391 | *Plectropomus leopardus* | Nov-2013 | Helix | Pomacentridae | *Neopomacentrus azysron* | yellow-tail demoiselle |
| 364393 | *Plectropomus leopardus* | Nov-2013 | Helix | Clupeidae | *Sardinella longiceps* | Indian oil sardine |
| 364393 | *Plectropomus leopardus* | Nov-2013 | Helix | Caesionidae | *Pterocaesio digramma* | double-lined fusilier |
| 364393 | *Plectropomus leopardus* | Nov-2013 | Helix | Lethrinidae | *Lethrinus miniatus* | trumpet emperor |
| 364354 | *Plectropomus leopardus* | Nov-2013 | Helix | Pomacentridae | *Acanthochromis polyacanthus* | spiny chromis |
| 364354 | *Plectropomus leopardus* | Nov-2013 | Helix | Apogonidae | *Cheilodipterus quinquelineatus* | five-lined cardinalfish |
| 364361 | *Plectropomus leopardus* | Nov-2013 | Helix | Pomacentridae | *Pomacentrus moluccensis* | lemon damsel |
| 364361 | *Plectropomus leopardus* | Nov-2013 | Helix | Caesionidae | *Pterocaesio digramma* | double-lined fusilier |
| 346159 | *Plectropomus leopardus* | Nov-2013 | Helix | Pomacentridae | *Pomacentrus moluccensis* | lemon damsel |
| 346159 | *Plectropomus leopardus* | Nov-2013 | Helix | Labridae | *Cirrhilabrus punctatus* | dotted wrasse |
| 346159 | *Plectropomus leopardus* | Nov-2013 | Helix | Pomacentridae | *Neopomacentrus azysron* | yellow-tail demoiselle |
| 346159 | *Plectropomus leopardus* | Nov-2013 | Helix | Lethrinidae | *Lethrinus miniatus* | trumpet emperor |
| 346160 | *Plectropomus leopardus* | Nov-2013 | Helix | Caesionidae | *Pterocaesio digramma* | double-lined fusilier |
| 346160 | *Plectropomus leopardus* | Nov-2013 | Helix | Pomacentridae | *Neopomacentrus azysron* | yellow-tail demoiselle |
| 346160 | *Plectropomus leopardus* | Nov-2013 | Helix | Lethrinidae | *Lethrinus miniatus* | trumpet emperor |
| 8367 | *Plectropomus leopardus* | Feb-2014 | Orpheus | Serranidae | *Cephalopholis boenak* | chocolate hind |
| 351218 | *Plectropomus leopardus* | May-2014 | Orpheus | Clupeidae | *Sardinella lemuru* | Bali sardinella |
| 351204 | *Plectropomus leopardus* | May-2014 | Orpheus | Caesionidae | *Caesio cuning* | redbelly yellowtail fusilier |
| 351204 | *Plectropomus leopardus* | May-2014 | Orpheus | Apogonidae | *Pristicon trimaculatus* | three-spot cardinalfish |
| 351213 | *Plectropomus leopardus* | May-2014 | Orpheus | Lethrinidae | *Lethrinus laticaudis* | grass emperor |
| 351206 | *Plectropomus leopardus* | May-2014 | Orpheus | Pomacentridae | *Neopomacentrus azysron* | yellow-tail demoiselle |
| 351209 | *Plectropomus leopardus* | May-2014 | Orpheus | Pomacentridae | *Neopomacentrus azysron* | yellow-tail demoiselle |
| 351205 | *Plectropomus leopardus* | May-2014 | Orpheus | Pomacentridae | *Pomacentrus trichrourus* | paletail damsel |
| 351205 | *Plectropomus leopardus* | May-2014 | Orpheus | Labridae | *Cirrhilabrus punctatus* | dotted wrasse |
| 351205 | *Plectropomus leopardus* | May-2014 | Orpheus | Caesionidae | *Pterocaesio digramma* | double-lined fusilier |
| 351205 | *Plectropomus leopardus* | May-2014 | Orpheus | Pomacentridae | *Neopomacentrus azysron* | yellow-tail demoiselle |
| 351216 | *Plectropomus leopardus* | May-2014 | Orpheus | Clupeidae | *Sardinella lemuru* | Bali sardinella |
| 351216 | *Plectropomus leopardus* | May-2014 | Orpheus | Pomacentridae | *Acanthochromis polyacanthus* | spiny chromis |
| 351216 | *Plectropomus leopardus* | May-2014 | Orpheus | Caesionidae | *Caesio caerulaurea* | blue and gold fusilier |
| 351216 | *Plectropomus leopardus* | May-2014 | Orpheus | Caesionidae | *Pterocaesio digramma* | double-lined fusilier |
| 351216 | *Plectropomus leopardus* | May-2014 | Orpheus | Caesionidae | *Pterocaesio tile* | dark-banded fusilier |
| 351216 | *Plectropomus leopardus* | May-2014 | Orpheus | Pomacentridae | *Neopomacentrus azysron* | yellow-tail demoiselle |
| 351216 | *Plectropomus leopardus* | May-2014 | Orpheus | Lethrinidae | *Lethrinus laticaudis* | grass emperor |
| 345534 | *Plectropomus leopardus* | Sep-2013 | Orpheus | Labridae | *Scarus sp.* | parrotfish |
| 8380 | *Plectropomus maculatus* | Dec-2013 | Orpheus | Pomacentridae | *Neopomacentrus azysron* | yellow-tail demoiselle |
| 8380 | *Plectropomus maculatus* | Dec-2013 | Orpheus | Serranidae | *Cephalopholis boenak* | chocolate hind |
| 351215 | *Plectropomus maculatus* | May-2014 | Orpheus | Labridae | *Leptoscarus vaigiensis* | marbled parrotfish |
| 351215 | *Plectropomus maculatus* | May-2014 | Orpheus | Pomacentridae | *Neopomacentrus azysron* | yellow-tail demoiselle |
| 351219 | *Plectropomus maculatus* | May-2014 | Orpheus | Caesionidae | *Pterocaesio digramma* | double-lined fusilier |
| 351211 | *Plectropomus maculatus* | May-2014 | Orpheus | Caesionidae | *Pterocaesio digramma* | double-lined fusilier |
| 351211 | *Plectropomus maculatus* | May-2014 | Orpheus | Pomacentridae | *Neopomacentrus azysron* | yellow-tail demoiselle |
| 351202 | *Plectropomus maculatus* | May-2014 | Orpheus | Pomacentridae | *Neopomacentrus azysron* | yellow-tail demoiselle |
| 351202 | *Plectropomus maculatus* | May-2014 | Orpheus | Lethrinidae | *Lethrinus nebulosus* | spangled emperor |
| 351202 | *Plectropomus maculatus* | May-2014 | Orpheus | Lethrinidae | *Lethrinus laticaudis* | grass emperor |
| 351212 | *Plectropomus maculatus* | May-2014 | Orpheus | Labridae | *Leptoscarus vaigiensis* | marbled parrotfish |
| 351212 | *Plectropomus maculatus* | May-2014 | Orpheus | Pomacentridae | *Pomacentrus trichrourus* | paletail damsel |
| 351212 | *Plectropomus maculatus* | May-2014 | Orpheus | Pomacentridae | *Neopomacentrus azysron* | yellow-tail demoiselle |
| 351214 | *Plectropomus maculatus* | May-2014 | Orpheus | Caesionidae | *Pterocaesio digramma* | double-lined fusilier |
| 351214 | *Plectropomus maculatus* | May-2014 | Orpheus | Pomacentridae | *Neopomacentrus azysron* | yellow-tail demoiselle |
| 351214 | *Plectropomus maculatus* | May-2014 | Orpheus | Synodontidae | *Synodus variegatus* | variegated lizardfish |
| 351214 | *Plectropomus maculatus* | May-2014 | Orpheus | Lethrinidae | *Lethrinus laticaudis* | grass emperor |
| 351203 | *Plectropomus maculatus* | May-2014 | Orpheus | Nemipteridae | *Scolopsis monogramma* | monogrammed monocle bream |
| 351203 | *Plectropomus maculatus* | May-2014 | Orpheus | Lethrinidae | *Lethrinus lentjan* | pink ear emperor |
| 345540 | *Plectropomus maculatus* | Sep-2013 | Orpheus | Gobiidae | *Amblygobius sphynx* | sphinx goby |
| 345540 | *Plectropomus maculatus* | Sep-2013 | Orpheus | Gobiidae | *Eviota ancora* | goby |
| 345540 | *Plectropomus maculatus* | Sep-2013 | Orpheus | Pomacentridae | *Neopomacentrus azysron* | yellow-tail demoiselle |


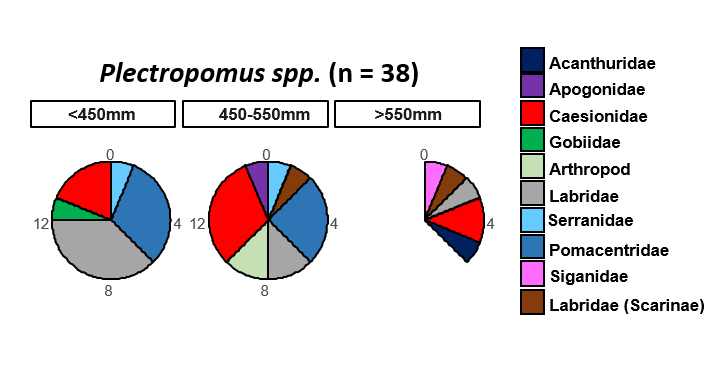


Supplementary Figure S1: Composition of prey (family-level) from visual stomach content analysis grouped by *Plectropomus* size-classes. All species and samples were pooled due to low amount of stomachs with identifiable prey. The indicated sample size refers to the number of prey items.


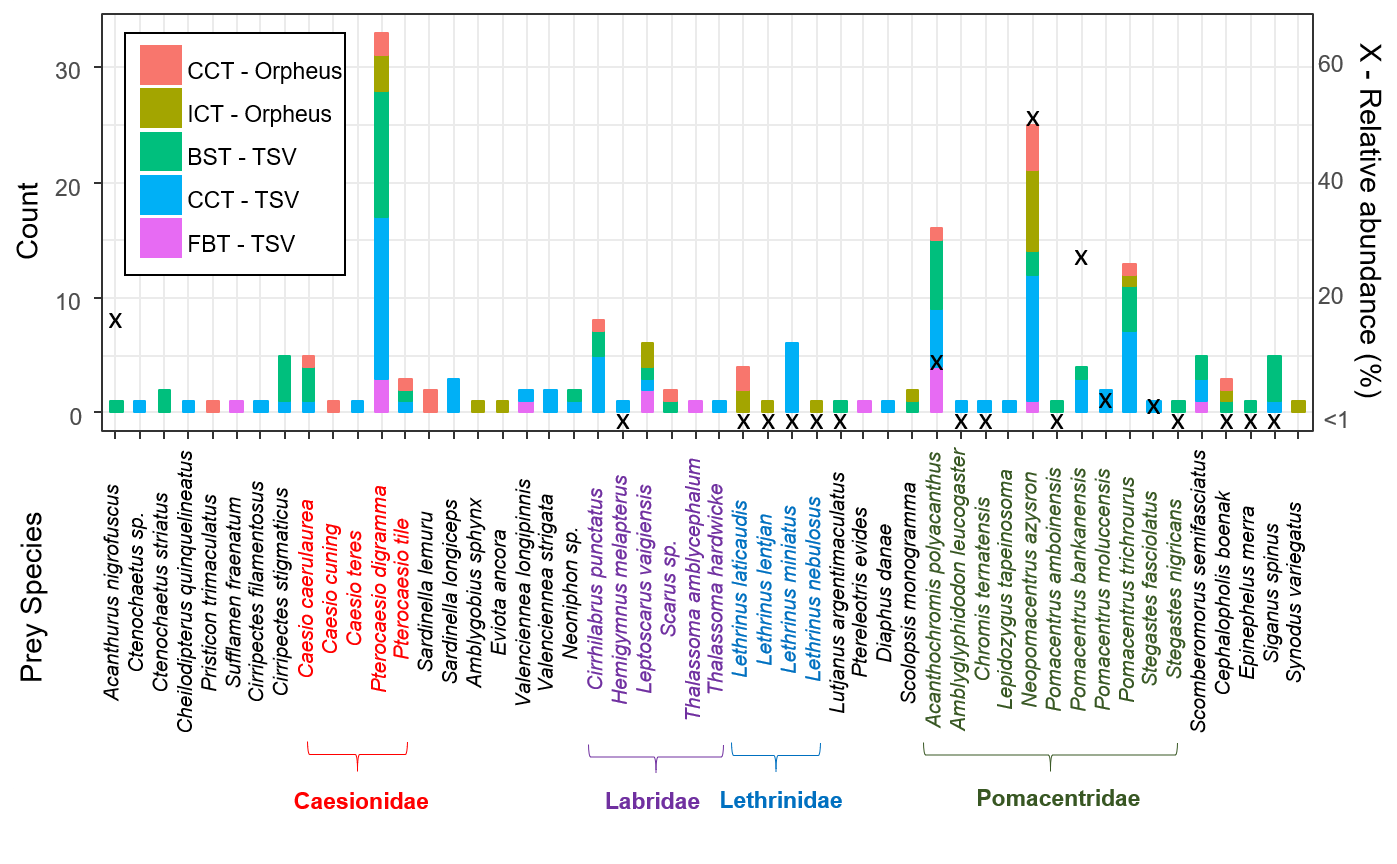


Supplementary Figure S2: Summary of diet composition from DNA stomach contents of each species/colour phase and main sampling area (all TSV reefs and sampling periods were combined). A secondary axis is included on right side indicating relative abundances (indicated with an ‘x’) of available prey species obtained by Bierwagen et al. - *in review*. The most abundant prey families of *Plectropomus* (i.e., Caesionidae, Pomacentridae, Labridae, Lethrinidae) are identified for informational purposes.


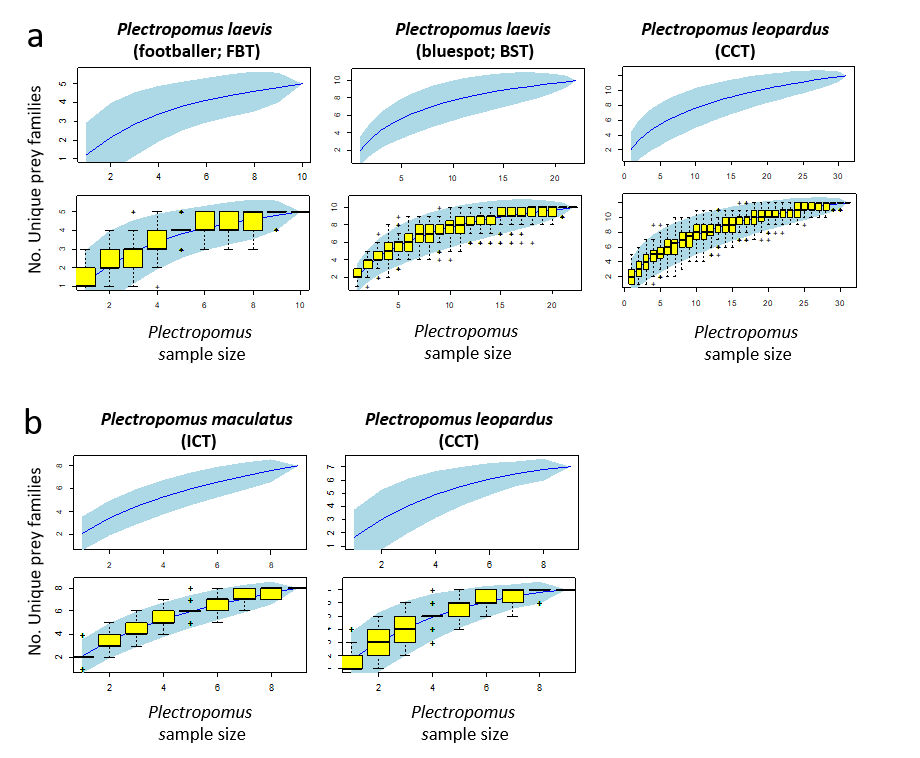


Supplementary Figure S3: Cumulative prey (family-level) curves derived from DNA-based stomach contents using a randomized accumulation method fitted using a Lomolino nonlinear regression model. Sample size was considered adequate when curves approached an asymptote. 95% confidence intervals are shaded in blue. Boxplots are included in the lower plots of each species/colour phase.


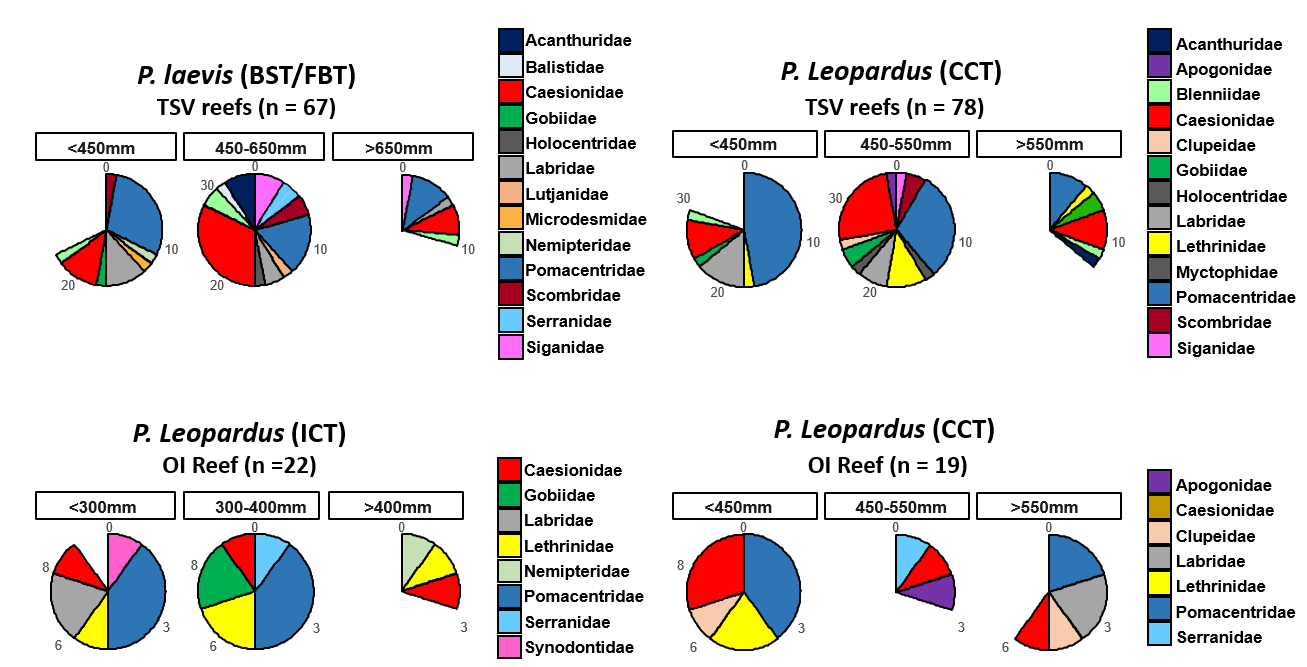


Supplementary Figure S4: Composition of prey (family-level) from DNA stomach content analysis grouped by *Plectropomus* size-classes. All samples from TSV reefs were pooled for respective species. The indicated sample size refers to the number of prey items.


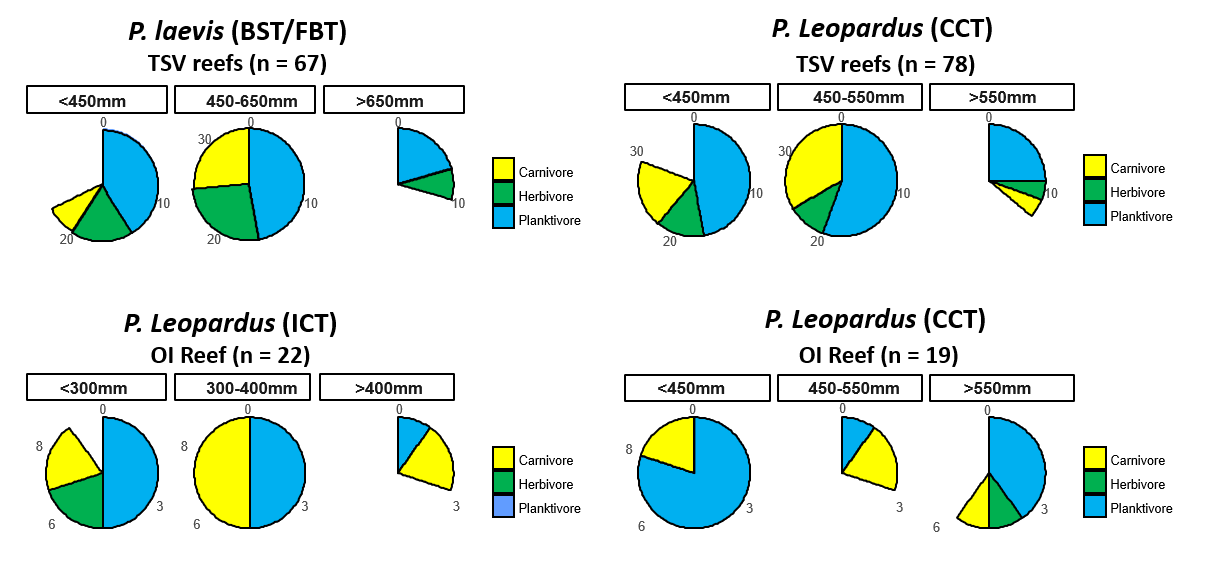


Supplementary Figure S5: Composition of prey functional modes from DNA stomach content analysis grouped by *Plectropomus* size-classes. All samples from TSV reefs were pooled for respective species. The indicated sample size refers to the number of prey items.


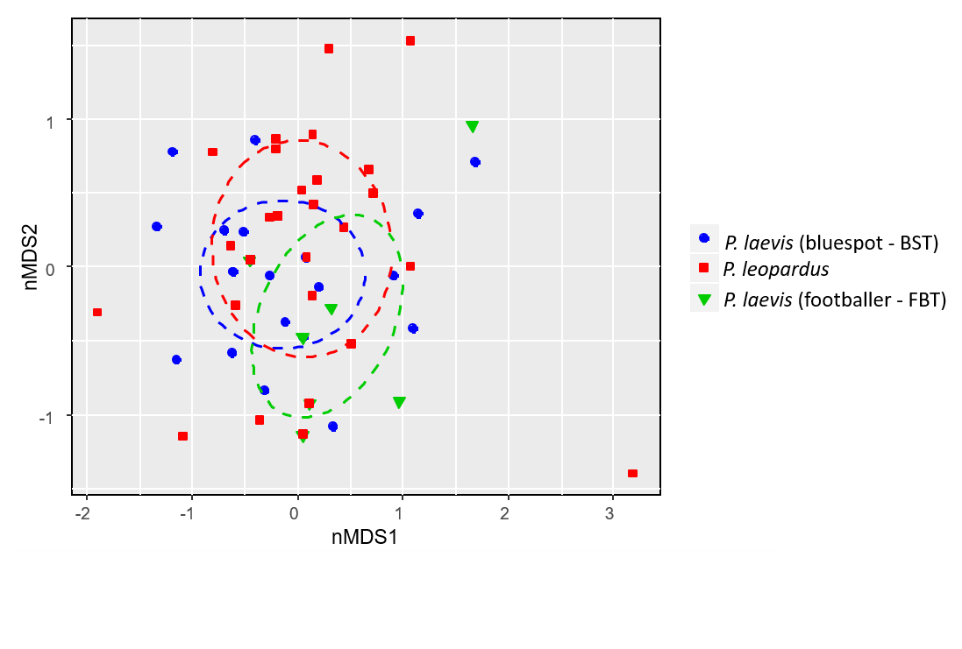


Supplementary Figure S6: Nonmetric multidimensional scaling (nMDS) plot characterising DNA stomach analysis relative to prey species of *Plectropomus* spp. at TSV reefs (Helix, Yankee, and Coil Reefs. A two-dimensional Bray-Curtis dissimilarity index was used resulting in a stress level of 0.08, ANOSIM R-statistic of 0.04, and P-value of 0.15. Prey species that only occurred once in all stomachs were removed to ensure model convergence.

**
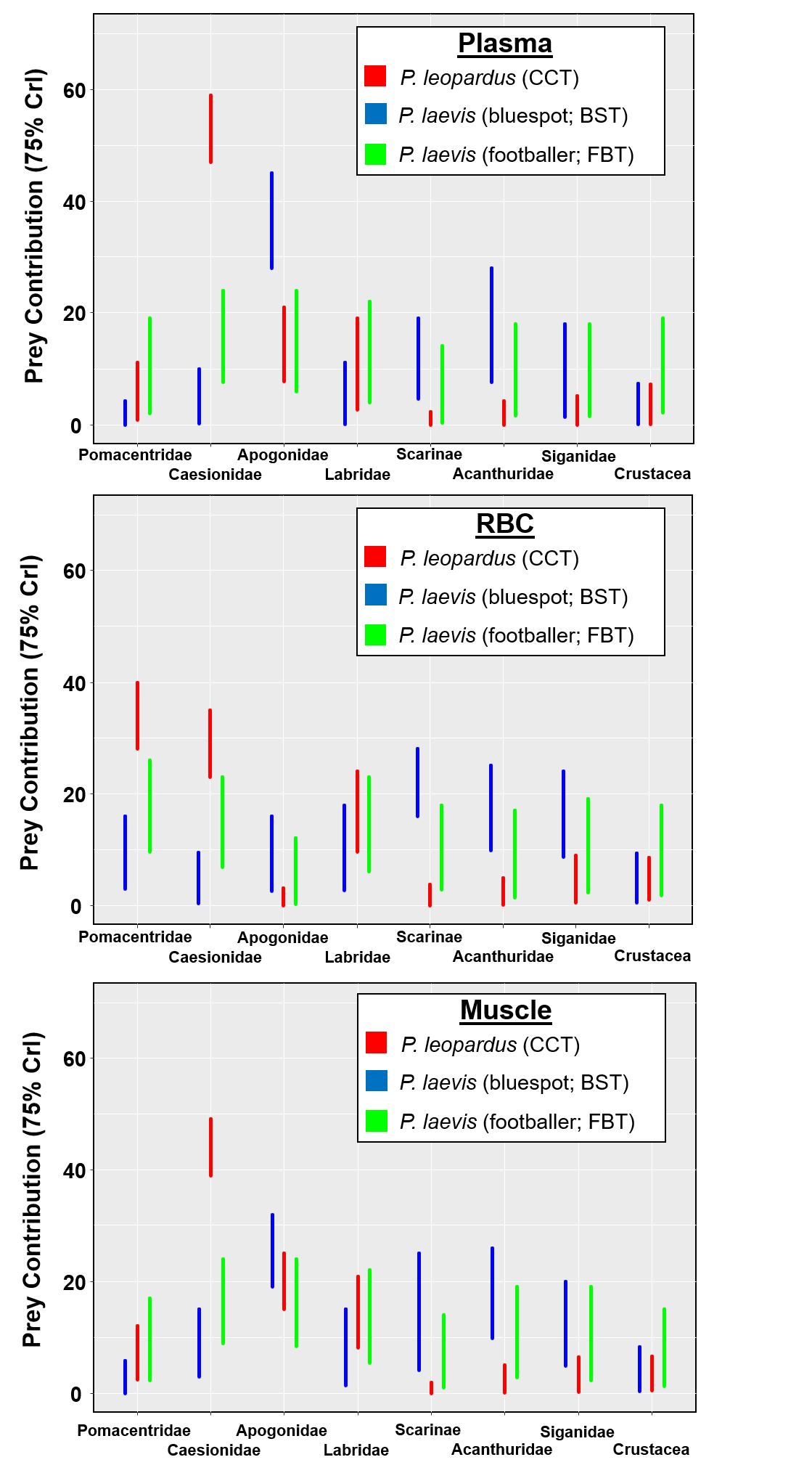
**

Supplementary Figure S7: Prey contribution estimates (75% credibility intervals) for *Plectropomus* spp. at TSV reefs (Helix, Yankee, and Coil Reefs combined) based on the SIAR package Bayesian stable isotope mixing models (adjusted for plasma, RBC, and muscle discrimination factors, respectively (Matley et al. 2016a)) without using priors from DNA gut content analysis. Caesionidae were correlated with Pomacentridae and Serranidae in the diagnostic matrix plot indicating the exact proportion of contribution may vary among these prey. Serranidae was removed from this analysis because it contributed <5% of DNA gut contents. Caesionidae was also correlated to Apogonidae for *P. leopardus* – muscle and *P. laevis* (bluespot) – RBC.

**
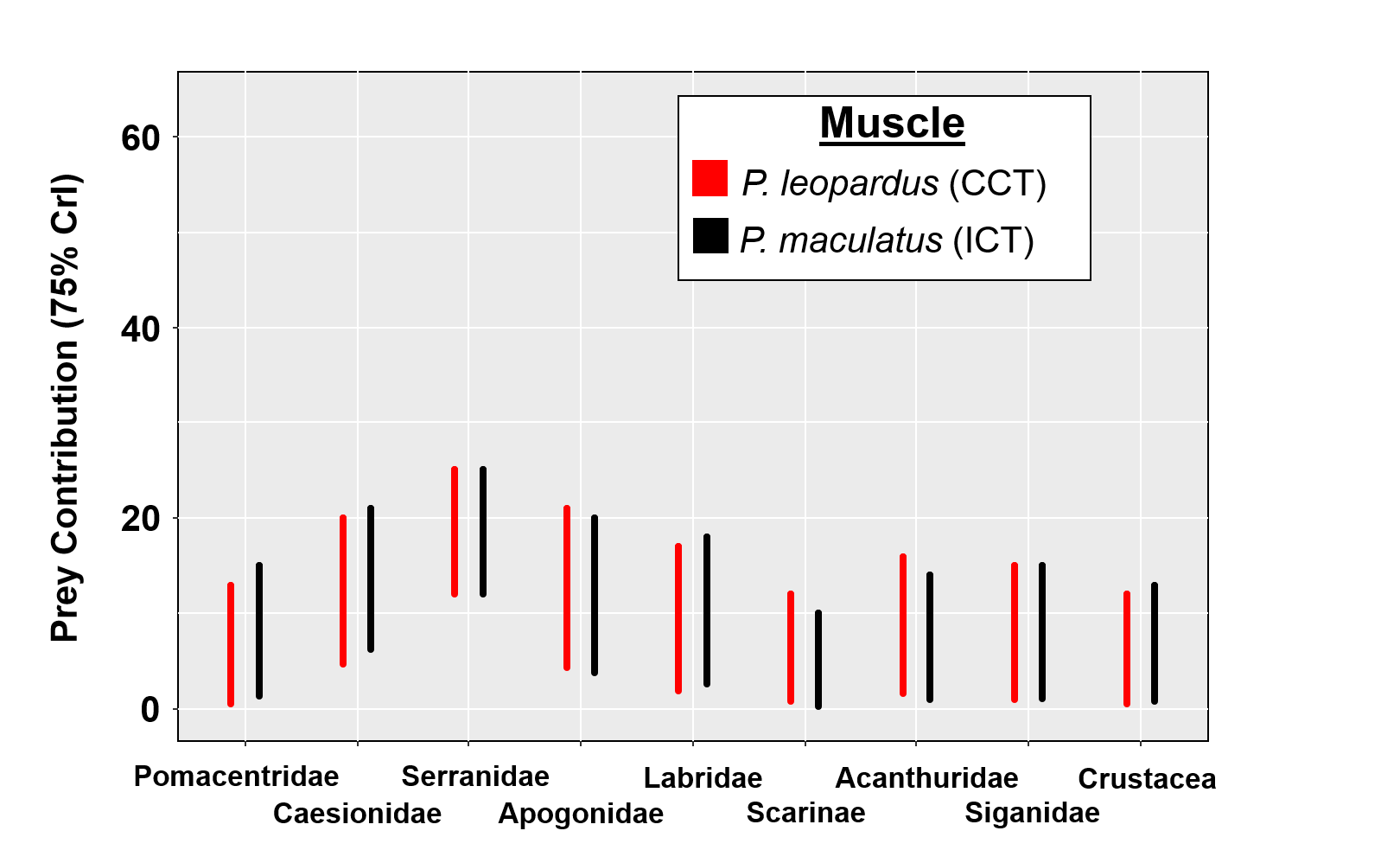
**

Supplementary Figure S8: Prey contribution estimates (75% credibility intervals) for *Plectropomus* spp. at Orpheus Island Reef based on the SIAR package Bayesian stable isotope mixing models (adjusted for muscle discrimination factors (Matley et al. 2016a)) without using priors from DNA gut content analysis. Caesionidae were correlated with Serranidae in the diagnostic matrix plot indicating the exact proportion of contribution may vary among these prey.
